# Mechanical control of innate immune responses against viral infection revealed in a human lung alveolus chip

**DOI:** 10.1038/s41467-022-29562-4

**Published:** 2022-04-08

**Authors:** Haiqing Bai, Longlong Si, Amanda Jiang, Chaitra Belgur, Yunhao Zhai, Roberto Plebani, Crystal Yuri Oh, Melissa Rodas, Aditya Patil, Atiq Nurani, Sarah E. Gilpin, Rani K. Powers, Girija Goyal, Rachelle Prantil-Baun, Donald E. Ingber

**Affiliations:** 1 Wyss Institute for Biologically Inspired Engineering, Harvard University, Boston, MA 02115 USA; 2 Vascular Biology Program and Department of Surgery, Boston Children’s Hospital, Harvard Medical School, Boston, MA 02115 USA; 3 Center on Advanced Studies and Technology (CAST), Department of Medical, Oral and Biotechnological Sciences, “G. d’Annunzio” University of Chieti-Pescara, Chieti, 66023 Italy; 4 Harvard John A. Paulson School of Engineering and Applied Sciences, Cambridge, MA 02138 USA

**Keywords:** Tissue engineering, Viral infection, Innate immunity, Antimicrobial responses

## Abstract

Mechanical breathing motions have a fundamental function in lung development and disease, but little is known about how they contribute to host innate immunity. Here we use a human lung alveolus chip that experiences cyclic breathing-like deformations to investigate whether physical forces influence innate immune responses to viral infection. Influenza H3N2 infection of mechanically active chips induces a cascade of host responses including increased lung permeability, apoptosis, cell regeneration, cytokines production, and recruitment of circulating immune cells. Comparison with static chips reveals that breathing motions suppress viral replication by activating protective innate immune responses in epithelial and endothelial cells, which are mediated in part through activation of the mechanosensitive ion channel TRPV4 and signaling via receptor for advanced glycation end products (RAGE). RAGE inhibitors suppress cytokines induction, while TRPV4 inhibition attenuates both inflammation and viral burden, in infected chips with breathing motions. Therefore, TRPV4 and RAGE may serve as new targets for therapeutic intervention in patients infected with influenza and other potential pandemic viruses that cause life-threatening lung inflammation.

## Abstract (web summary)

Mechanical forces in lungs facilitate breathing motions. Here the authors use a microfluidic human lung alveolus chip to study influenza infection and find that mechanical forces from active chips also induce innate inflammatory responses via, at least partially, signaling from TRPV4 and RAGE, thereby implicating them as potential therapeutic targets for lung inflammation.

## Introduction

Innate immune responses in the lung serve as a first line of defense against infections by respiratory viruses, such as influenza A viruses and SARS-CoV-2, yet we know little about how these responses are controlled locally within the physical microenvironment of the human breathing lung. One reason is that it is difficult, if not impossible, to tease out the regulatory cues in the local environment within a living organ in vivo, and the mechanical cues encountered in the human lung are absent in conventional in vitro culture or organoid models.

Breathing is central to life, but its biological effects in lung beyond oxygenation are poorly understood. Breathing motions in human lung exert dynamic physical forces on its constituent cells and tissues that promote lung branching^1^ and alveolar differentiation^2^ during normal lung development, and contribute to the etiology of various lung diseases, including acute lung injury^3,4^, pulmonary edema^5^, and pulmonary fibrosis^6^. Mechanical forces have been implicated in activation of neutrophils, monocytes, and macrophages as well^7^. However, nothing is known about how breathing motions and mechanical forces impact innate immunity and responses to infection within lung parenchymal cells and pulmonary microvascular endothelium in an organ-relevant context. This is in large part because of the lack of experimental models that can mimic human lung structures and pathophysiology at the tissue and organ levels while permitting control over breathing motions.

Influenza A viruses represent an ongoing threat to public health due to rapid mutation and reassortment of the viral genome. H3N2 strain is one of the predominant influenza strains in circulation and a leading cause of lower respiratory infection and hospitalization^8^. H3N2 infection in the lung alveolus damages the tissue barrier (alveolar-endothelial interface), induces pulmonary edema, recruits inflammatory cells, and if uncontrolled, causes severe viral pneumonia and acute respiratory distress syndrome (ARDS), often leading to death^9^. Current studies of influenza infection in the distal lung rely on human cell lines that fail to recapitulate complex disease pathogenesis in vivo or mouse models that do not reflect the anatomy or pathophysiology of the natural human host.

To overcome these limitations, human microphysiological systems, such as human organ-on-a-chip (Organ Chip) microfluidic devices have been developed that culture human cells in organ-specific three-dimensional environments to imitate the complex chemical, structural, and physical cues seen in vivo. The alveolus chip recapitulates the human alveolar-capillary interface with an air-liquid interface (ALI) and vascular fluid flow while allowing independent control over breathing motions^4,5,10^. Previously we showed that the alveolus chip can faithfully mimic human lung physiology, disease states, and adenoviral vector-mediated gene therapy delivery, as well as therapeutic efficacy and toxicity responses^4,5,10,11,12^. However, it is unknown if this model can accurately replicate human lung responses to influenza viral infection and explore the role of breathing motions during disease progression.

Here we use an optimized human alveolus chip to model H3N2 influenza infection in vitro. We report that breathing motions suppress viral replication through a novel mechano-chemical control mechanism that involves the transient receptor potential vanilloid-type 4 (*TRPV4*) and the receptor for advanced glycation end products (*RAGE*). Targeting these molecular mediators influences viral burden and host inflammation on-chip. Thus, human alveolus chip represents a clinically relevant platform for preclinical disease modeling and drug testing, thereby expediting therapeutic development against respiratory infections.

## Results

### Recapitulation of alveolar cell differentiation and alveolar-capillary interface formation

**Fig. 1:**
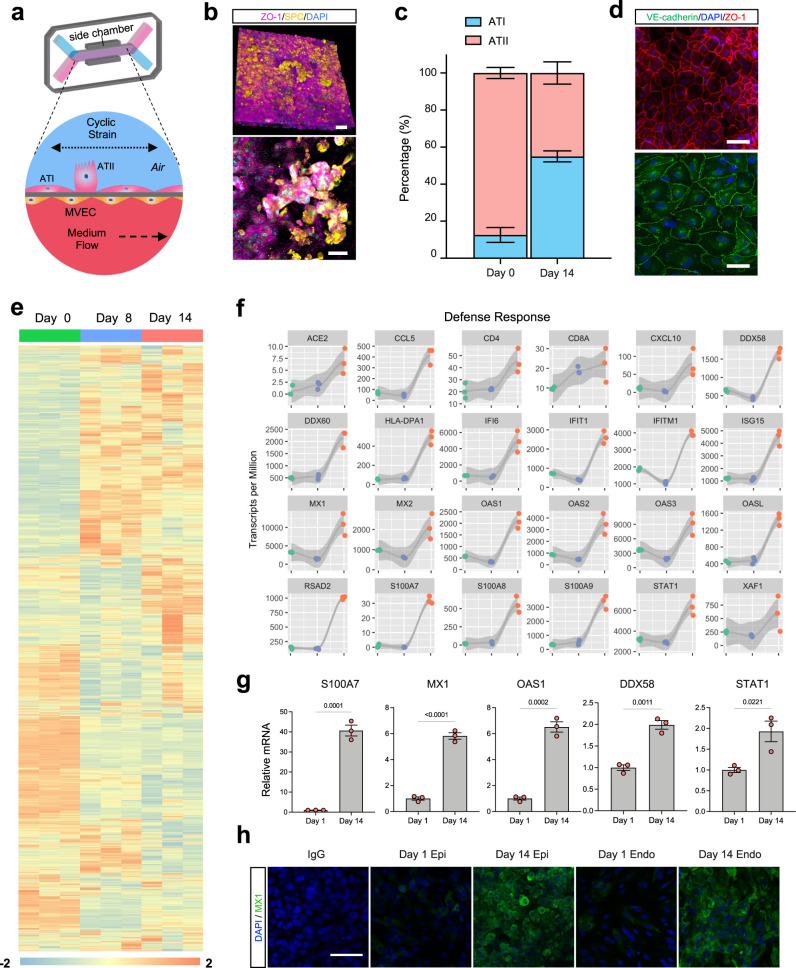
The Human alveolus chip. **a** Schematic of human alveolus chip with primary alveolar epithelial type I (ATI) and type II (ATII) cells lining the upper surface of the porous ECM-coated membrane in the air channel with and pulmonary microvascular endothelial cells (MVEC) on the lower surface of the same membrane in the basal vascular channel that is continuously perfused with medium. The entire membrane and adherent alveolar-capillary interface are exposed to physiological cyclic strain by applying cyclic suction to neighboring hollow chambers (gray) within the flexible PDMS microfluidic device. **b** Two magnifications of immunofluorescence micrographs showing the distribution of ZO1-containing tight junctions and ATII cell marker surfactant protein C (*SPC*) in the epithelium of the alveolus chip (bar, 50 μm). **c** Graph showing the percentages of ATI and ATII cells at the time of plating and 14 days after culture on-chip. Data represent mean ± SD; *n* = 3 biological chip replicates. **d** Immunofluorescence micrographs showing alveolar epithelial cells (top) and endothelial cells (bottom) within the alveolus chip stained for ZO1 and VE-cadherin, respectively (scale bar, 50 μm). **e** Temporal gene expression profiles in the alveolar epithelial cells on-chip. **f** Smoothed regressions of time course showing expression of selected genes that are involved in host defense response in epithelial cells cultured for up to 2 weeks on-chip; *n* = 3 biological chip replicates. Gray zone indicates the 95% confidence interval for predictions from a linear model; green, day 0; blue, day 8; pink, day 14. **g** Graph showing the mRNA levels of key genes from **f** in the epithelial cells of alveolus chips at day 1 and day 14 after culture. Data represent mean ± SD, *n* = 3 biological chip replicates independent of the RNA-seq samples. Unpaired two-tailed *t*-test. **h** Immunofluorescence staining showing increased MX1 expression in both alveolar epithelial cells and microvascular endothelial cells on-chip at day 14 compared to day 1 of culture. Scale bar: 50 μm. Source data are provided as a Source Data file.

The human alveolus chip used in this study is a microfluidic device containing two parallel channels separated by an extracellular matrix (ECM)-coated porous membrane lined by primary human lung alveolar epithelium cells cultured under an ALI on its upper surface and primary human pulmonary microvascular endothelial cells on the lower surface, which are fed by continuous flow of culture medium through the lumen of the lower vascular channel (Fig. 1a)^10,12,13^. The engineered alveolar-capillary interface is also exposed to cyclic mechanical deformations (5% cyclic strain at 0.25 Hz) to mimic physiological breathing motions at tidal volume^14^ and the normal respiratory rate of humans via application of cyclic suction to hollow side chambers within the flexible polydimethylsiloxane (PDMS) device (Fig. 1a).

Using a commercial source of primary human lung alveolar epithelial cells composed of 90% alveolar type II (ATII) cells (Supplementary Fig. 1a) and an optimized ECM coating that resembles the composition of basement membranes in vivo^15^ (200 µg/ml collagen IV and 15 µg/ml laminin), we found that the alveolus chips support differentiation of the ATII cells into to alveolar type I (ATI) cells (Fig. 1b and Supplementary Fig. 1b) resulting in a final ratio of ATII to ATI cells of 55:45 as demonstrated by immunostaining of ATII cell marker surfactant protein C (SPC) and the ATI cell marker RAGE (Fig. 1c). This is also accompanied by formation of a tight epithelial barrier (Supplementary Fig. 1c), which approximates that observed in vivo^16^. Confluent monolayers of interfaced alveolar epithelium and endothelium also display continuous cell-cell junctions stained apically with zonula occludens-1 (ZO-1) in epithelium and laterally with vascular endothelial-cadherin (VE-cadherin), respectively, by 14 days of culture (Fig. 1d), which are maintained for at least 5 weeks on-chip.

RNA-sequencing (RNA-seq) analysis of the alveolar epithelium showed that epithelial cells cultured on-chip exhibit extensive changes in mRNA expression over 14 days in culture (Fig. 1e) and display distinct transcriptome changes compared to the same cells maintained in conventional static Transwell cultures, as demonstrated by principal component analysis (PCA) and differential gene expression analysis (Supplementary Fig. 2a, b). Among the top differentially expressed genes (DEGs) are genes related to multicellular organismal homeostasis, respiratory tube development, and vasculature development, which is consistent with the establishment of epithelial-endothelial signaling crosstalk required for the maturation of the alveolar epithelium and the microvascular endothelium (Supplementary Fig. 2c and Supplementary Data 1 and 2) as we observed on-chip (Fig. 1).

Using short time-series expression miner (STEM)^17^, we identified five gene expression patterns that exhibited statistically significant changes over time when cultured on-chip in the presence of breathing-like motions (Fig. 1e and Supplementary Fig. 3a). Gene ontology (GO) analysis revealed that many of the upregulated genes are involved in biological processes relevant to differentiation, including anatomical structure development, cell adhesion, movement of cell or subcellular components, and ECM organization (Supplementary Fig. 3b). Examples include aquaporin 5 (*AQP5*) and podoplanin (*PDPN*) (Supplementary Fig. 4a) that are known markers for ATI cells^18^, confirming the differentiation from ATII to ATI cells that we observed by immunostaining (Fig. 1b and Supplementary Fig. 1b). Lipoprotein lipase (*LPL*) and surfactant protein D (*SFTPD*), two genes involved in surfactant production, also increased (Supplementary Fig. 4a), consistent with the previous finding that mechanical strain promotes enhanced surfactant secretion in vivo^19^ and on-chip^4^. The downregulated pathways (profiles 0 and 4, Supplementary Fig. 3b) are associated with cell junctions and ECM organization, which include genes such as *CLDN11* (claudin 11), *CDH5* (cadherin 5), and *PLAT* (plasminogen activator) (Supplementary Fig. 3c).

The upregulation of several ion channels and transporters (Supplementary Fig. 4b) suggests that alveolar fluid clearance, which is essential for maintenance of air-liquid interface, may be increased as well. Many genes belonging to defense responses also were upregulated from day 8 to 14 (Fig. 1f), including angiotensin converting enzyme 2 (*ACE2*), the receptor for SARS-CoV-2, which is also a key mediator of lung physiology and pathophysiology^20^. Other important innate immune response genes include myxovirus resistance 1 (*MX1)*, 2′−5′-oligoadenylate synthetase 1 (*OAS1*), DExD/H-Box Helicase 58 (*DDX58*), *s*ignal transducer and activator of transcription 1 (*STAT1*), which we confirmed using qPCR (Fig. 1g) and immunofluorescence microscopy (Fig. 1h). Importantly, this upregulation of genes related to alveolar development and defense response is also seen in human lung during the transition from fetus to birth (Supplementary Data 3)^21,22^.

Significant genetic reprogramming was also observed in endothelial cells cultured in the presence of cyclic mechanical strain on-chip over 14 days in culture (Supplementary Fig. 5a, b). The Wnt signaling pathway is essential for cross-talk between endothelium and epithelium during lung development^23^ and consistent with this, we found a number of Wnt ligands, including *WNT3*, *WNT7A*, *WNT7B*, *WNT9A*, and *WNT10A*, exhibit elevated expression in endothelial cells on-chip in the presence of breathing motions (Supplementary Fig. 5c). Together, our analysis reveals that human lung alveolus chips that are exposed to physiological breathing motions recapitulate perinatal maturation of the alveolar-capillary interface, cell-ECM interactions, and epithelial-endothelial crosstalk that are indispensable for the development, homeostasis, and regeneration of the lung alveolus^24^.

### Influenza A virus infection of the human lung alveolus chip

**Fig. 2:**
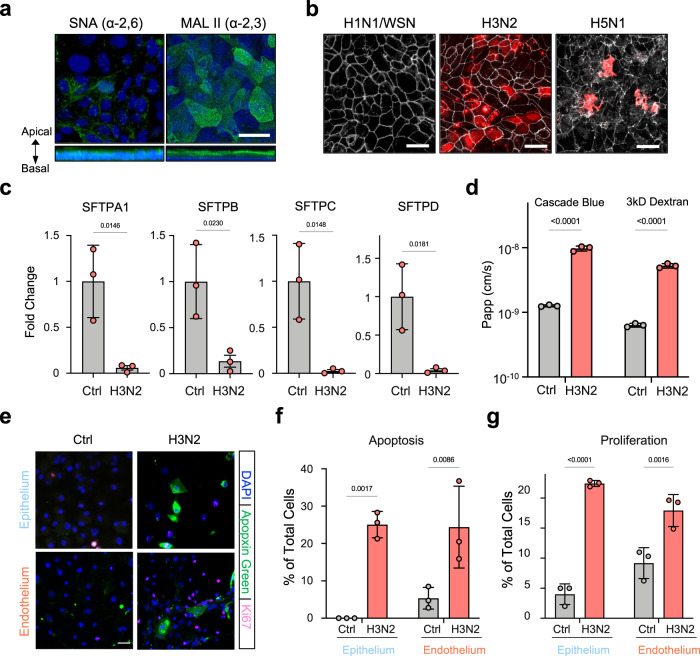
Cellular phenotyping after influenza A virus infection of the human alveolus chip. **a** Top and side fluorescence views of the alveolar epithelium cultured on-chip and stained for α−2,3-linked sialic acid or α−2,6-linked sialic acid using Maackia Amurensis Lectin II (MAL II) and Sambucus nigra agglutinin (SNA), respectively (bar, 50 µm). **b** Top fluorescence views of the alveolar epithelium on-chip infected with three different influenza virus strains (WSN (H1N1), HK/68 (H3N2), or HK/97 (H5N1) at MOI = 1) and stained for viral nuclear protein (NP) in red and ZO-1 in white. Scale bar, 50 µm. **c** Graph showing decreased mRNA levels of surfactant genes by HK/68 (H3N2) virus infection at MOI = 1 compared with uninfected as control (Ctrl). **d** Graph showing increased lung permeability to cascade blue and 3 kD dextran by H3N2 infection on-chip. **e** Immunofluorescent images showing increased apoptosis as indicated by Apopxin Green staining and cell proliferation as indicated by Ki67 staining (bar, 50 µm). **f**, **g** Graph showing quantifications of cell apoptosis (**f**) and proliferation (**g**) at 24 h after H3N2 infection on-chip. For **c**, **d**, **f**, **g**, data represent mean ± SD.; *n* = 3 biological chip replicates; unpaired two-tailed *t*-test. Source data are provided as a Source Data file.

Influenza A virus entry is mediated by hemagglutinin binding to sialic acid receptor on host cells, with avian and mammalian viral strains preferentially binding to α−2,3- or α−2,6-linked sialylated glycans, respectively^25^. Using glycan-specific lectins, we found that the human lung alveolar epithelial cells predominantly express α−2,3-linked sialic acid receptors on their apical surface on-chip (Fig. 2a). Consistent with this finding and results obtained in human lung tissues in vivo^26^, we found that influenza A/HongKong/8/68 (HK/68; H3N2) and A/HongKong/156/1997 (HK/97; H5N1) viruses successfully infect the epithelium in the human alveolus chip, whereas the influenza A/WSN/33 (WSN; H1N1) virus does not, as detected by immunostaining of viral nuclear protein (NP) (Fig. 2b). The specificity of this response is further exemplified by the finding that H1N1 virus that preferentially infects large airways also infects human Lung Airway Chips lined by bronchial epithelium much more effectively than H3N2^27^.

Consistent with the observation that influenza A virus mainly target ATII cells in the human lung^28^, we found that H3N2 infection results in over 90% reduction of the surfactant gene expression (Fig. 2c). This is also consistent with recent observation that SARS-CoV-2 infection in the alveolus leads to suppression of the ATII program^29^. Loss of ATII cells caused by H3N2 infection leads to increased lung permeability (Fig. 2d) as well as a 25% increase in apoptotic cells as indicated by Apopxin Green staining (Fig. 2e, f), while no necrosis was observed at 48 h after infection. The absence of necrosis-mediated cell death by H3N2 infection on-chip is consistent with reports from human biopsies indicating that this type of cell death is associated with more severe human and avian infections caused by H5N1 and H9N2 strains^30^. We also observed a significant increase in cell proliferation by quantifying Ki67 staining (Fig. 2e, g), indicating the initiation of a repair program similar to that observed in vivo^31^. Similar injury and repair responses are also observed in the endothelial cells (Fig. 2e–g), supporting recent evidence that lung endothelium possesses substantial regenerative capacity after viral pneumonia^32^. In summary, our alveolus chip replicates both the viral and cellular tropisms and cellular phenotypical alterations that are known to be produced by influenza infection in the human distal lung, including loss of barrier, cell death, and regeneration.

### Multi-level host responses to infection in the human lung alveolus chip

**Fig. 3:**
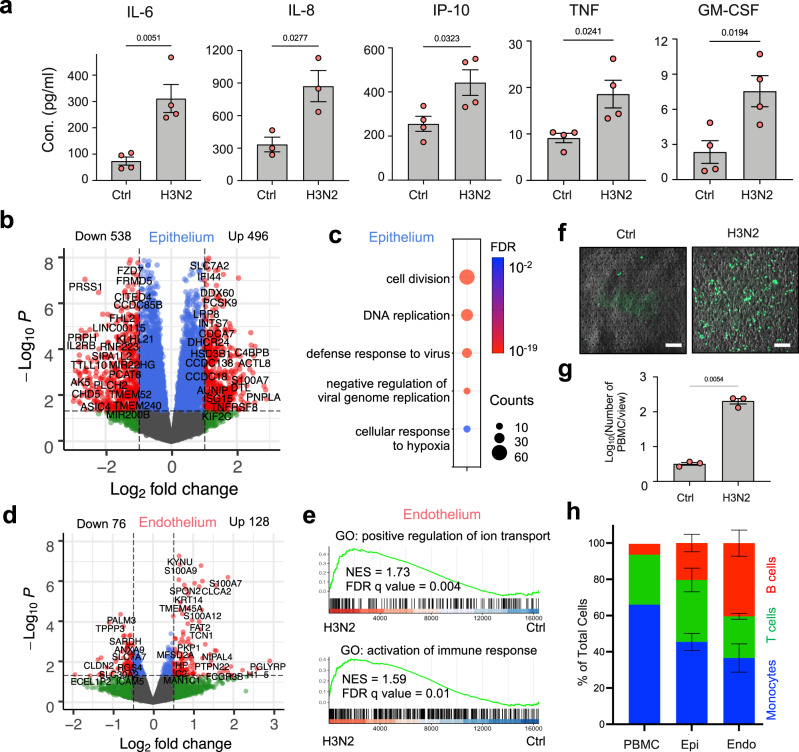
Systematic inflammation after influenza A virus infection of the human alveolus chip. **a** Concentrations of the indicated cytokines at day 17 measured by Luminex assay in the effluent of the vascular channels of alveolus chips infected with HK/68 (H3N2) virus (MOI = 1) versus control untreated chips (Ctrl) at 48 hpi in the presence of 5% strain. Data represent mean ± SD; *n* = 4 biological chip replicates except for IL-8 (*n* = 3) from two independent experiments; unpaired two-tailed *t*-test. **b** Volcano plot of DEGs in epithelial cells from HK/68 (H3N2)-infected alveolus chips (MOI = 1) compared to control uninfected chips. *P* values were adjusted using Bonferroni correction for multiple comparisons. **c** Dot plot visualization of enriched biological processes in epithelial cells of HK/68 (H3N2) infected alveolus chips (MOI = 1). **d** Volcano plot of DEGs in endothelial cells from HK/68 (H3N2)-infected alveolus chips (MOI = 1) compared to control uninfected chips. *P* values were adjusted using Bonferroni correction for multiple comparisons. **e** Gene Set Enrichment Analysis (GSEA) plots showing the significant enrichment of two gene sets in endothelial cells from HK/68 (H3N2)-infected (MOI = 1) compared with control uninfected alveolus chips. **f** Fluorescence imaging for CellTracker Green-labeled PBMCs at the endothelial cell surface 2 hours after perfusion under static conditions through the vascular channel of uninfected control (Ctrl) versus HK/68 (H3N2)-infected alveolus chips at 24 hpi (MOI = 1). Scale bar: 100 µm. **g** Graph showing number of PBMCs recruited to the endothelium in response to infection by HK/68 (H3N2) (MOI = 1) and the baseline level of PBMCs in uninfected chips (Ctrl). Data represent mean ± SD. *n* = 3 biological chip replicates; unpaired two-tailed *t*-test. **h** Graph showing the relative percentages of monocytes, T cells, and B cells before being added to the chips or in the epithelial (epi) or endothelial cell channel (endo) 2 h after being added to endothelial channel of the chips. Data represent mean ± SD.; *n* = 3 biological chip replicates. Source data are provided as a Source Data file.

As cytokine production by lung epithelial and endothelial cells is a key feature of early host innate immune response to viral infections, we analyzed cytokines that are secreted into the basal vascular outflow using Luminex assays, analogous to measurement of plasma cytokine levels in vivo. These studies revealed that infection with influenza H3N2 virus induces significantly higher levels of interleukin 6 (IL-6), IL-8, interferon gamma-induced protein 10 (IP-10), tumor necrosis factor alpha (TNF), and granulocyte-macrophage colony-stimulating factor (GM-CSF) in the vascular outflows at 48 h post infection (hpi) compared to control uninfected chips (Fig. 3a). RNA-seq of the epithelial cells carried out at the same time point (48 hpi) revealed 496 upregulated genes and 538 downregulated genes (Fig. 3b). GO analysis indicated that the differentially expressed genes (DEGs) belong to biological pathways including cell division and DNA replication that may be associated with antiviral defense responses and activation of alveolar barrier repair in response to virus-induced injury (Fig. 3c and Supplementary Fig. 6a), which is consistent with the observation of increased Ki67^+^ proliferative cells (Fig. 2f). Some of these DEGs, such as *CXCL10* (C-X-C Motif Chemokine Ligand 10, encodes IP-10) and *IL6*, were further confirmed by qPCR (Supplementary Fig. 6b).

A more detailed analysis of antiviral responses induced in the lung epithelium in the alveolus chip revealed a dominant type III interferon (IFN-III) response mediated by interferon lambda (IFNλ) (Supplementary Fig. 6c). Importantly, this finding differs from results obtained from analysis of human lung alveolar epithelial cells cultured in static 2D culture on rigid planar dishes where IFNβ-mediated type I interferon response prevails^33^. However, our results replicate in vivo findings^34^, which suggest that IFN-III mediates the front-line response to viral infection in mucosal tissues^35^. In contrast to the epithelium (Fig. 3b), a more limited effect on the host transcriptome was observed in the endothelium (Fig. 3d). But enrichment analysis shows that genes involved in activation of immune responses and ion transport are also upregulated in these cells (Fig. 3e). Interestingly, however, this is likely through tissue-tissue signaling crosstalk as viral mRNA could not be detected within the endothelium.

Influenza virus infection also upregulates expression of endothelial adhesion molecules, thereby allowing the recruitment of leukocytes to the alveolus in vivo^9^. When fluorescently labeled primary human peripheral blood mononuclear cells (PBMCs) were flowed through the endothelium-lined vascular channel 24 hpi with influenza H3N2, we detected upregulation of ICAM1 and TNF in the endothelial cells by qPCR, indicating endothelial inflammation (Supplementary Fig. 6d). Indeed, this resulted in more than a 100-fold increase in the adhesion of the circulating PBMCs to the surface of the activated endothelium compared to control uninfected chips (Fig. 3f, g and Supplementary Fig. 6e). To determine which immune cell types are recruited, we isolated human CD14^+^ monocytes, CD3^+^ T cells, and CD19^+^ B cells, labeled each population with a different fluorescent cell tracker, mix them at a ratio of 6:3:1 (monocytes: T cells: B cells), and perfused the mixture through the vascular channel of the chip 24 h after infection (Supplementary Fig. 6f). Two hours after perfusion under static conditions, we found that all of the cell types were able to adhere to the endothelium or transmigrate up into the epithelium (Supplementary Fig. 6g and Fig. 3h). Interestingly, a higher percentage of B cells remained in the endothelium than T cells, which is in agreement with the timing of T cell activation preceding that of B cells. In addition, the recruitment of immune cells further increased the levels of cytokines, such as IL-8, IL-18, and S100 Calcium Binding Protein A8 and A9 (S100A8/9; also named as Calprotectin) (Supplementary Fig. 6h), consistent with our previous findings from the human Airway Chip^36^. Thus, the human breathing alveolus chip faithfully replicates multi-level host innate immune responses of lung alveoli to influenza A virus infection.

### Breathing-like mechanical deformations suppress viral infection on-chip

**Fig. 4:**
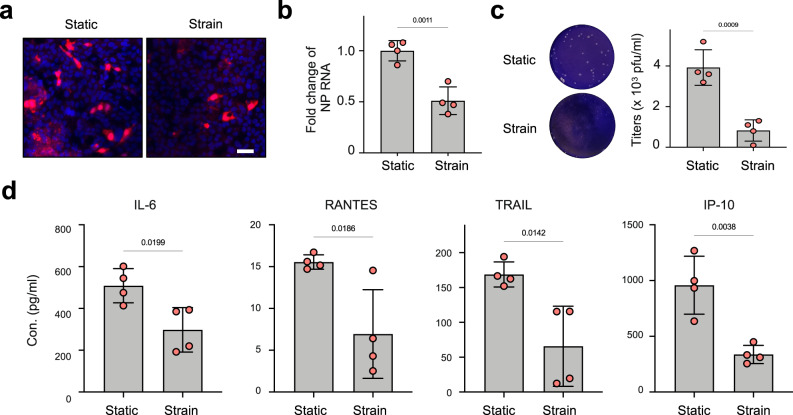
Cyclic mechanical strain inhibits viral infection in alveolus chip. **a** Immunostaining of influenza virus NP (red) in alveolus chips that are cultured under static conditions (Static) or under 5% and 0.25 Hz cyclic mechanical strains (Strain) for 48 h and then infected with HK/68 (H3N2) (MOI = 1) for another 48 h (blue, DAPI-stained nuclei; scale bar, 50 µm). **b** Graph showing fold changes in RNA levels of influenza virus NP in the epithelium within alveolus chips from **a** as measured by qPCR. Data represent mean ± SD; *n* = 4 biological chip replicates; unpaired two-tailed *t*-test. **c** Images (left) and graph (right) showing plaque titers of virus in the apical washes of alveolus chips from **a**. Data represent mean ± SD; *n* = 4 biological chip replicates; unpaired two-tailed *t*-test. **d** Graphs showing cytokine production in the vascular effluents of alveolus chips from **a** at 48 hpi. Data represent mean ± SD; *n* = 4 biological chip replicates; unpaired two-tailed *t*-test. Source data are provided as a Source Data file.

We next explored the role of cyclic respiratory motions in virus-induced respiratory infections by infecting alveolus chips with H3N2 virus on day 15 of culture and measuring viral loads in the presence or absence of physiological, breathing-like, mechanical deformations (5% strain, 0.25 Hz). Immunostaining for influenza virus nucleoprotein (NP) revealed significant suppression of viral infection in lung alveolar epithelial cells exposed to breathing motions 2 days following the introduction of virus on-chip compared to static chip controls (Fig. 4a). This inhibition by mechanical stimulation was further confirmed using qPCR and a plaque assay, which respectively show that the application of cyclic mechanical strain leads to a 50% reduction of viral mRNA in the epithelium (Fig. 4b) and ~80% reduction of viral titers in apical washes compared to static controls (Fig. 4c). This also was accompanied by a reduction in the production of the inflammatory cytokines, IL-6, RANTES (regulated on activation, normal T cell expressed and secreted), TRAIL (tumor necrosis factor-related apoptosis-inducing ligand or Apo 2 ligand), and IP-10, as measured in the vascular outflows from the chips (Fig. 4d). Moreover, similar inhibition of viral infection by cyclic mechanical strain was observed when the alveolus chips were infected with other respiratory viruses, including H5N1 influenza virus (Supplementary Fig. 7a) and the common cold OC43 coronavirus (Supplementary Fig. 7b).

### Mechanical strain increases innate immunity in Lung Chips

To gain insight into how mechanical strain associated with physiological breathing motions might normally act to combat viral infection, we analyzed the RNA-seq analysis time course and noticed that exposure to continuous cyclic mechanical deformations resulted in increased expression of multiple IFN-related antiviral genes, including *DDX58*, *MX1*, *OAS1*, and *STAT1*, in both lung epithelial and endothelial cells from day 8 to day 14 of culture (Fig. 1f, g and Supplementary Fig. 5b). Indeed, many interferon-stimulated genes (ISGs) have higher expression in mechanically stimulated cells at day 14 on-chip but not in cells cultured in parallel in static Transwells (Supplementary Fig. 8a), and this was confirmed by qPCR analysis which showed increased expression of type I and type III interferons (Supplementary Fig. 8b).

**Fig. 5:**
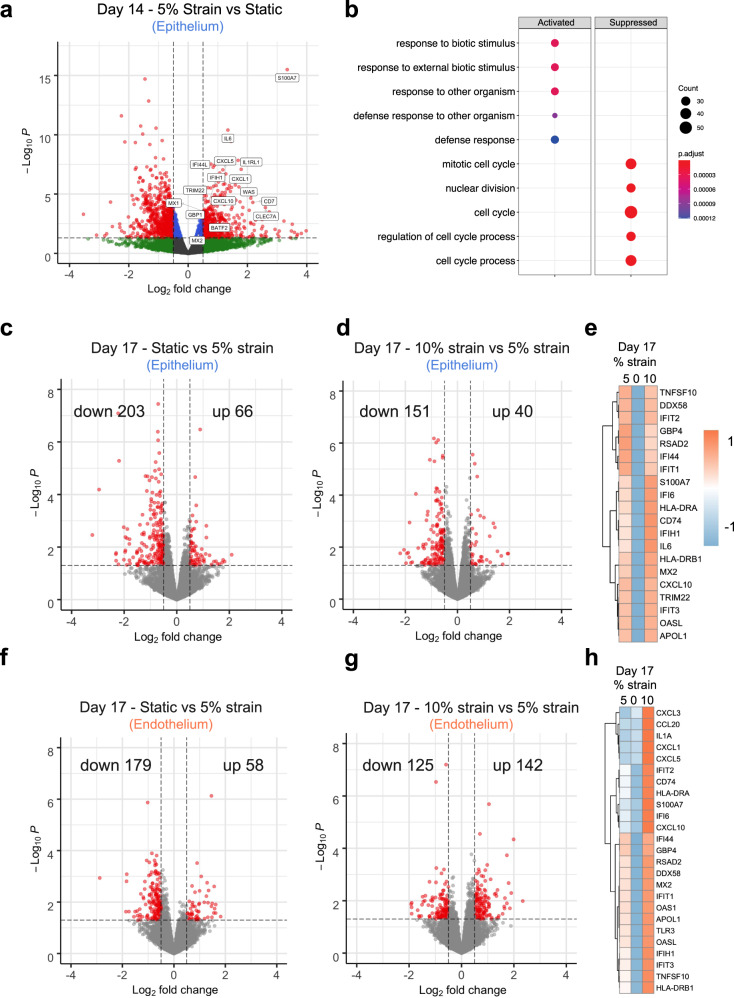
Mechanical strain reversibly regulates innate immune response in alveolus chip in uninfected chips. **a** Volcano plot of DEGs comparing epithelial cells from alveolus chips under static or 5% strain culture condition for 4 days. DEGs (*P*_adj_ < 0.05) with a fold change >1.5 (or <−1.5) are indicated in red. The names of DEGs belonging to the innate immune pathway are labeled. **b** Dot plot showing the biological processes activated or suppressed by 5% strain vs. static culture condition in alveolus chips from **a**. **c**, **d** Volcano plots of DEGs showing the effects of switching 5% strain to static for 2 days on epithelial cells on-chip (**c**) and the effects of increasing 5% strain to 10% strain for 2 days on epithelial cells on-chip (**d**). **e** Heat map showing differentially expressed innate immune genes in epithelial cells under different magnitudes of mechanical strains. **f**, **g** Volcano plots of DEGs showing the effects of switching 5% strain to static for 2 days on endothelial cells on-chip (**f**) and the effects of increasing 5% strain to 10% strain for 2 days on endothelial cells on-chip (**g**). **h** Heat map showing differentially expressed innate immune genes in endothelial cells under different magnitudes of mechanical strains. *P* values were adjusted using Bonferroni correction for multiple comparisons in **a**–**d**, **f**, and **g**.

To directly determine whether mechanical strain influences innate immunity pathway signaling, we cultured the alveolus chips in the presence or absence of cyclic mechanical strain from day 10 to 14 and performed RNA-seq analysis. Consistent with our earlier results, lung alveolar epithelial cells mechanically stimulated on-chip exhibit higher expression of ISGs and cytokines, such as *MX1*, *MX2*, *IL6*, *CXCL10*, *CXCL5* (C-X-C motif chemokine ligand 5), *IFI44L* (interferon induced protein 44 like), and *IFIH1* (interferon induced with helicase C domain 1), compared to static chip controls (Fig. 5a). Functional enrichment analysis of DEGs demonstrated that application of physiological cyclic strain activates pathways related to host defense response, while suppressing processes related to cell cycle and cell proliferation (Fig. 5b).

To further investigate the association between mechanical strain and innate immunity, we switched a subset of chips that had been exposed to 5% cyclic mechanical strain from day 10 to 14 to static conditions or to elevated mechanical strain (10%) condition on day 15 while continuing to mechanically stimulate the remaining chips at 5% for 2 additional days. The choice of 10% strain is based on our calculation^37–39^ (Supplementary Data 4) that both low-tidal volume (6 ml/kg) and high-tidal volume ventilation (12 ml/kg) can lead to ~10% mechanical strain in the alveolus, which is often seen in patients with emphysema or lung edema due to increased lung compliance or surface tension^40^. RNA-seq analysis of the epithelial cells on day 17 revealed that cessation of mechanical stimulation results in suppression of the innate immune response, exemplified by decreased expression of antiviral genes, such as *IFIT1* (interferon-induced protein with tetratricopeptide repeats 1), *IFIT2*, *OASL* (2′−5′-Oligoadenylate Synthetase Like), and *DDX58* (Fig. 5c and Supplementary Fig. 8c). On the other hand, elevating the level of mechanical stimulation to 10% strain results in modest upregulation of innate immune response genes (Fig. 5d, e and Supplementary Fig. 8d). In parallel, similar reduction in innate immune response pathway signaling is observed in endothelial cells when mechanical stimulation ceases (Fig. 5f and Supplementary Fig. 8e). However, increasing mechanical strain to 10% results in further upregulation of innate immune response genes (Fig. 5g, h and Supplementary Fig. 8f), including several inflammatory cytokines, such as *CXCL3* (C-X-C motif chemokine ligand 3), *CCL20* (C-C motif chemokine ligand 20), *IL1A* (interleukin-1 alpha), *CXCL1* (C-X-C motif chemokine ligand 1), and *CXCL5* (Fig. 5h). These results suggest that endothelial cells may be more sensitive to elevated mechanical strains than the epithelial cells. Together, these results demonstrate that cyclic mechanical forces similar to those experienced during breathing in the lung sustain higher levels of antiviral innate immunity in both lung alveolar epithelial cells and pulmonary microvascular endothelial cells compared cells cultured in the same chips under static conditions, which helps to explain why viral infection efficiency is reduced in the mechanically strained alveolus chips.

### S100A7 mediates the effects of mechanical strain on lung innate immunity

**Fig. 6:**
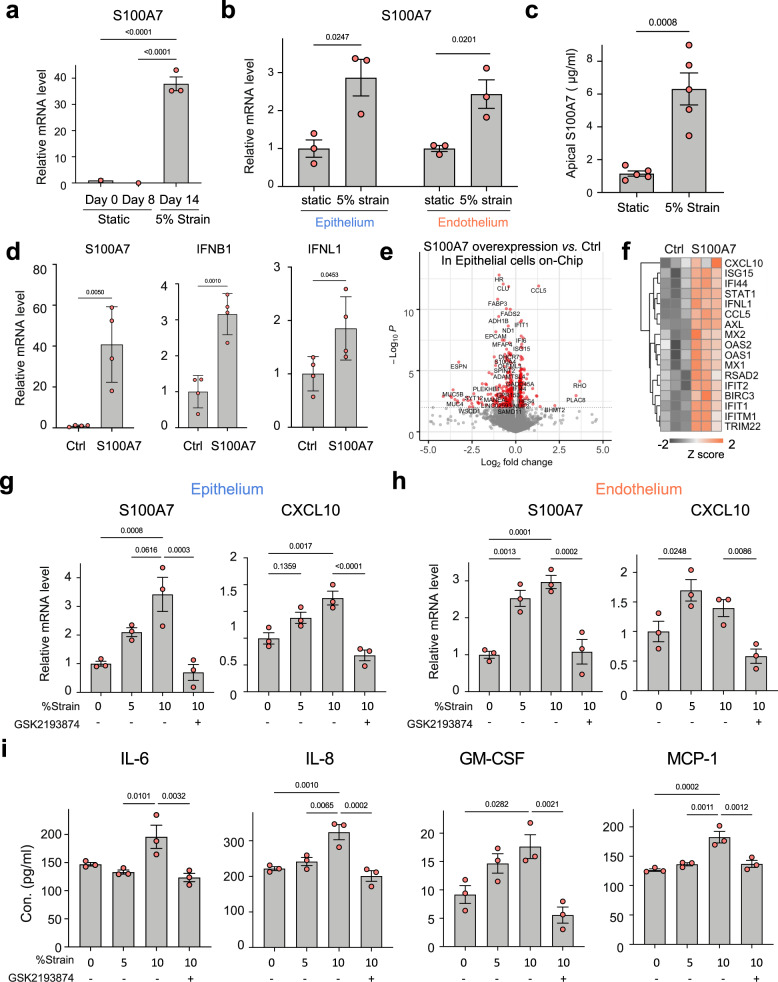
Mechanical strain-induced S100A7 increases innate immunity. **a** Graph showing the levels of S100A7 mRNA (transcripts per million) in the epithelial cells of alveolus chips at different time points of culture, measured by RNA-seq. Data represent mean ± SD; *n* = 3 biological chip replicates; one-way ANOVA with Bonferroni multiple comparisons test. **b** Graph showing the mRNA level of S100A7 in epithelial cells or in endothelial cells when cultured under 5% strain or under static conditions for 4 days (day 10–14) on-chip. Data are shown as mean ± SD; *n* = 3 biological chip replicates; unpaired two-tailed *t*-test. **c** Graph showing the protein levels of S100A7 in the apical washes of alveolus chips under strain or static condition, measure by ELISA. Data are shown as mean ± SEM; *n* = 5 biological chip replicates from 2 independent experiments; unpaired two-tailed *t*-test. **d** Graphs showing fold changes in mRNA levels of S100A7, IFNβ1, and IFNλ1 in epithelial cells of the alveolus chips that were transfected with human S100A7-expressing plasmid or the vector control (Con) for 48 h. Data are shown as mean ± SD; *n* = 4 biological chip replicates; unpaired two-tailed *t*-test. **e** Volcano plots of DEGs showing the effects of S100A7 overexpression on transcriptome in epithelial cells of the alveolus chips that were transfected with S100A7-expressing plasmid for 2 days with the empty plasmid as a control (Ctrl). *P* values were adjusted using Bonferroni correction for multiple comparisons. **f** Heat map showing that S100A7 upregulates the expression of many genes involved in innate immune response in epithelial cells of the alveolus chips. *n* = 3 biological chip replicates. Graphs showing the mRNA levels of S100A7 and CXCL10 in epithelial cells (**g**) and endothelial cells (**h**) and cytokine levels on chips cultured under static condition (0% strain), 5% strain, 10% strain, and 10% strain perfused with or without 1 µM TRPV4 inhibitor GSK2193874 for 48 h (**i**). Data in **g**–**i** represent mean ± SEM; *n* = 3 biological chip replicates; one-way ANOVA with Bonferroni multiple comparisons correction. Source data are provided as a Source Data file.

We then leveraged this Organ Chip approach to explore the underlying mechanism responsible for these effects by examining our RNA-seq data sets. This analysis revealed that the S100 calcium-binding protein A7 (*S100A7*), which encodes a member of the S100 family of alarmins proteins and a ligand for RAGE, as one of the top genes differentially expressed in response to cyclic strain application in all experiments (Figs. 1f and 5a, c, e, and Supplementary Fig. 9a), and one that was not induced cells in static culture on-chip or in Transwells (Fig. 6a and Supplementary Fig. 9a). Chips cultured under physiological 5% strain from day 10 to 14 exhibit significantly higher mRNA levels of S100A7 in both epithelium and endothelium than when the cells were cultured under static conditions (Fig. 6b). This increase in *S100A7* gene expression in response to mechanical stimulation is also associated with a significant increase in S100A7 protein secretion, as determined by quantifying its levels in apical washes from human alveolus chips using an enzyme-linked immunosorbent assay (ELISA) (Fig. 6c). Moreover, over-expression of S100A7 induces significant upregulation of the antiviral cytokines IFNβ1 and IFNλ1 in lung alveolar epithelial cells on-chip (Fig. 5d) as well as in conventional monoculture (Supplementary Fig. 9b) and in A549 lung cells, which similarly show both overexpression of S100A7 and reduced influenza H1N1 virus infection in a RAGE-dependent manner (Supplementary Fig. 9c, d). To directly examine the effect of S100A7 on gene transcriptional network, we performed RNA-seq analysis of alveolar epithelial cells on-chip after transfection of a plasmid expressing human *S100A7*. Differential gene expression analysis revealed that increased expression of S100A7 indeed upregulates many genes involved in the innate immune response, including *IFNL1*, *CCL5* (C-C motif chemokine ligand 5), *IFI6* (Interferon alpha-inducible protein 6), and *IL6* (Fig. 6e, f). Therefore, S100A7 that is induced by breathing motions is able to drive the host innate immune response.

### The mechanosensitive ion channel TRPV4 is upstream of S100A7

Cells can sense mechanical stimuli through diverse mechanosensitive molecules at the cell surface, including integrins, G protein coupled-receptors, growth factor receptors, and mechanosensitive ion channels (MSCs)^41^. The most rapid responses are mediated by MSCs that generate an ion flux in response to membrane deformation, and thereby regulate a wide variety of cellular processes, including innate immunity^42^. Mechanical strain has been reported to increase intracellular Ca^2+^ in lung alveolar epithelial cells and endothelial cells^43^, and pharmacological or genetic modulation of calcium flux through the mechanosensitive TRPV4 ion channel was found to regulate pulmonary barrier function in the lung alveolus chip^5,12^. Indeed, RNA-seq analysis confirmed that both cell types cultured in the lung alveolus chip express high level of *TRPV4* as well as other MSCs, such as piezo type mechanosensitive ion channel component 1 (*PIEZO1* and *PIEZO2* (Supplementary Fig. 10). Importantly, when we perfused the TRPV4 inhibitor, GSK2193874, through the vascular channel of the alveolus chip while exposing the alveolar-capillary interface to 10% mechanical strain at 0.25 Hz for 48 h, the upregulation of *S100A7* and *CXCL10* was suppressed as measured by PCR in both epithelial cells (Fig. 6g) and endothelial cells (Fig. 6h), and this was accompanied by a reduction in the levels the inflammatory cytokines IL-6, IL-8, GM-CSF, and MCP-1 (Monocyte chemoattractant protein-1) in the vascular effluent (Fig. 6i).

**Fig. 7:**
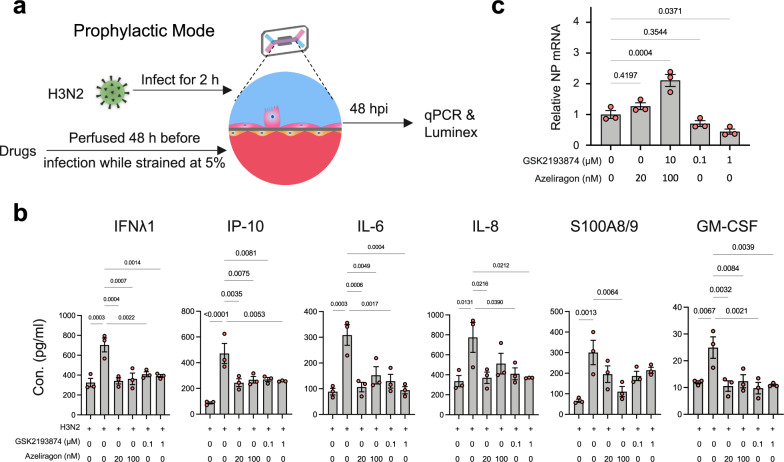
RAGE mediates mechanical strain-induced activation of innate immunity during infection. **a** Illustration of the experimental protocol involving prophylactic treatment with signaling inhibitors for 48 h prior to viral infection of the human alveolus chip. **b** Graphs showing cytokine levels on chips. **c** Graph showing mRNA levels of H3N2 NP in epithelial cells treated with azeliragon or GSK2193874 at indicated doses on-chip. Data in **b**, **c** represent mean ± SEM; *n* = 3 biological chip replicates; one-way ANOVA with Dunnett’s multiple comparisons correction. Source data are provided as a Source Data file.

To determine whether RAGE and TRPV4 mediate the effects of mechanical strain on innate immunity, we perfused the RAGE inhibitor azeliragon or the TRPV4 inhibitor GSK2193874 at non-toxic doses (Supplementary Fig. 11) through the vascular channel of the alveolus chip for 48 hours before infection with H3N2 while applying 5% mechanical strain (Fig. 7a). These studies confirmed that prophylactic inhibition of RAGE by pretreating the chips with either 20 or 100 nM azeliragon prevented the increase in cytokine production (Fig. 7b), although we observed that treatment with the higher dose resulted in a higher viral load (viral NP mRNA levels) when analyzed 48 h following infection (Fig. 7c). Interestingly, while pretreatment with the TRPV4 inhibitor also reduced cytokines, it decreased viral mRNA levels (Fig. 7b,c). This difference in viral load response suggests that TRPV4 is likely acting in multiple signaling pathways in parallel, and at least one other contributes to host response to viral infection via a distinct mechanism. In fact, prior work has shown that TRPV4 can influence viral infection by inducing nuclear translocation of DEAD-Box Helicase 3 X-Linked (DDX3X), a protein that promotes nuclear viral export and translation^44^. Taken together, these results confirm that TRPV4 is one of the players that mediate the mechanotransduction process by which mechanical strain applied to the lung alveolar epithelium and endothelium results in activation of S100A7/RAGE pathway, and thereby drives an innate immune response.

### RAGE inhibitors suppress viral infection-induced inflammatory responses

**Fig. 8:**
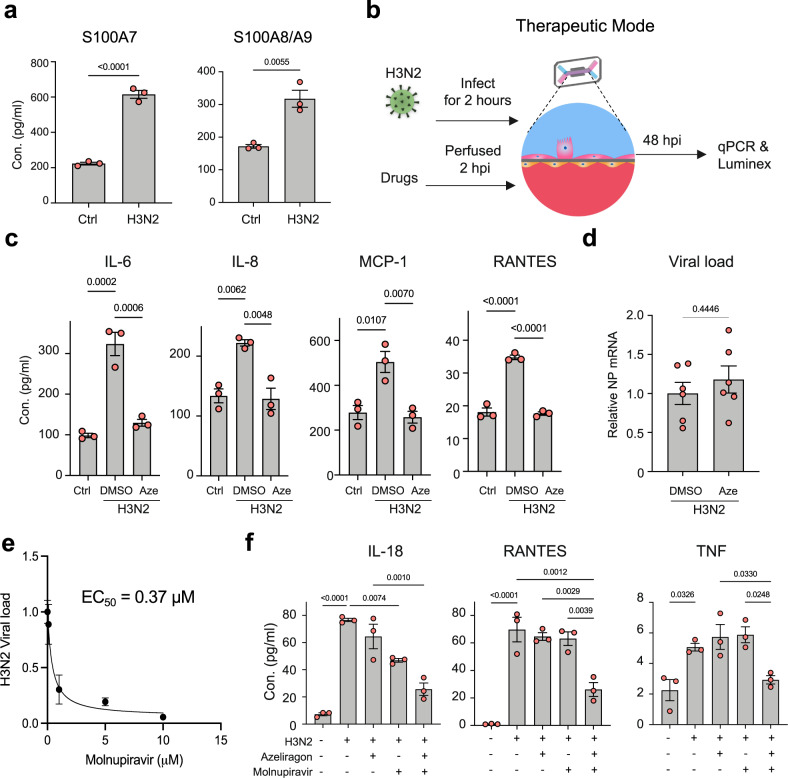
RAGE inhibitor azeliragon suppresses viral host inflammation responses and produces synergistic effects with molnupiravir when administered 2 h after infection. **a** Graph showing the protein levels of S100A7 (left) and S100A8/A9 (right) in the vascular effluents of the alveolus chips at 48 hpi with HK/68 (H3N2) virus (MOI = 1) in the presence of 5% strain. Data represent mean ± SD; *n* = 3 biological chip replicates; unpaired two-tailed *t*-test. **b** Illustration of drug study on the human alveolus chip. **c** Graphs showing the levels of cytokines in the vascular effluents of alveolus chips that were uninfected (Ctrl), or infected with HK/68 (H3N2) (MOI = 1) in the presence or absence of 100 nM Azeliragon (Aze). Data are shown as mean ± SD; *n* = 3 biological chip replicates; one-way ANOVA with Dunnett’s multiple comparisons test. **d** Graph showing that azeliragon (100 nM) had no effect on viral load when administered 2 h after infection. Data are shown as mean ± SEM; *n* = 6 biological chip replicates; unpaired two-tailed *t*-test. **e** Plot showing the effect of molnupiravir at different concentrations on H3N2 viral load determined on human Alveolus Transwell. Data are shown as mean ± SD; *n* = 2 biological replicates. EC50 (half maximal effective concentration) was determined by a variable slope fitting with four parameters. **f** Graphs showing that azeliragon (100 nM) and molnupiravir (500 nM) drug combo synergistically reduce the levels of cytokines in the vascular effluents of alveolus chips infected with H3N2 virus. Data are shown as mean ± SEM; *n* = 3 biological chip replicates; one-way ANOVA with Dunnett’s multiple comparisons test. Source data are provided as a Source Data file.

Interestingly, S100A7 gene expression and protein secretion are also upregulated in both epithelial cells and endothelial cells in response to infection of the alveolus chips with H3N2 influenza virus, and this is accompanied by upregulation of other S100 family members, including S100A8, S100A9, and S100A12 (Figs. 3b, d and 8a), which could further augment inflammation. As S100A7 and other members of the S100 family signal through RAGE^45^, we next explored whether RAGE inhibitors can be used to suppress host inflammatory responses during viral infection in alveolus chips when administered the drugs in a therapeutic mode (2 hours after infection). One advantage of human Organ Chips is that they permit drug testing in a more clinically relevant pharmacological setting by enabling clinically relevant doses of drugs (e.g., maximum blood concentration or *C*_max_) to be perfused through the vascular channel under flow as occurs in the vasculature of living organs^46^ (Fig. 8b). When azeliragon, a RAGE inhibitor that demonstrated safety but not efficacy in Phase III clinical trials involving more than 2000 patients with Alzheimer Disease and diabetic nephropathy, was perfused at its *C*_max_ at 100 nM^47^ through the vascular channel of human alveolus chips infected with H3N2 influenza virus while exposed to cyclic physiological (5%) strain, it significantly blocked induction of multiple cytokines, including IL-6, IL-8, IP-10 and RANTES (Fig. 8c), and similar effects are produced using a different RAGE inhibitor drug (FPS-ZM1) (Supplementary Fig. 12a). Interestingly, treatment with 100 nM azeliragon had no effect on viral load under these conditions (Fig. 8d), which is in contrast to when it was administered prophylactically (Fig. 7c). When we combined azeliragon with the potent ribonucleoside analog, molnupiravir, which demonstrates broad-spectrum antiviral activity against various RNA viruses, including influenza A (Fig. 8e), SARS-CoV-2, SARS-CoV, MERS-CoV, and Ebola^48,49^, we found that the combination produced synergistic inhibition of inflammatory cytokine production following H3N2 infection (Fig. 8f and Supplementary Fig. 12b), whereas there was no added effect on viral infection (Fig. 8d and Supplementary Fig. 12c). Taken together, these data support the therapeutic potential for RAGE inhibitors, such as azeliragon, as suppressors of host-damaging inflammatory responses to viral infection when used alone or in combination with direct antiviral drugs.

## Discussion

Here we describe how use of human Organ Chip technology has enabled discovery of a novel mechano-immunological control mechanism in which physiological breathing motions suppress viral infection in human lung alveoli by activating host innate immune responses. Exposure to cyclic mechanical cues associated with physiological breathing motions (5% strain; 15 deformations/min) sustains moderate levels of production and secretion of S100A7 protein by alveolar epithelium and endothelium, which binds to RAGE and generates an innate immune response that protects against viral infection, whereas S100A7 levels are lower and viral infection greater when breathing motions are absent. Our data also show that pharmacological inhibitors of RAGE signaling suppress inflammation during influenza infection, raising the possibility that RAGE inhibitors might represent novel adjuvants that may be used alone or in combination with antiviral therapies to suppress life-threatening inflammation of the distal airway that can be triggered by influenza A viruses. Importantly, they might also be useful for suppressing augmented inflammatory responses associated with conditions, such as COVID-19, which result in abnormally high levels of S100 family protein production^50,51^.

Lung alveoli are exposed to dynamic mechanical stresses with each breath and these physical deformations are known to influence lung development^2,52,53^, surfactant production^53,54^, and tissue barrier integrity^4^. Mechanical forces also have been suggested to activate immune cells in lung^55^, particularly in response to pathological stresses associated with hypeventilation^7,56^. But little is known about the effects of breathing-associated cyclic mechanical strain on host innate immunity in response to viral infections within the tissue parenchyma. Our study provides the first evidence indicating that physical forces control innate immunity in nonimmune cells and tissues, specifically in pulmonary alveolar epithelium and microvascular endothelium. This is important for the lung as it is exposed to numerous airborne antigens during early life while the adaptive immune system is still immature^57^. These findings are also supported by recent single-cell RNA-seq studies of lung alveolar epithelium, which demonstrate increased innate immunity at birth in both mouse and human^21,22^. In addition, our finding that a hyper-physiological deforming stress (10% strain) leads to an augmented host inflammatory response, especially in the endothelial cells, resonates with clinical findings, which show that low tidal volume ventilation that induces less cyclic mechanical strain results in lower plasma levels of inflammatory cytokines than use of a normal tidal volume strategy^58,59^; importantly, this greatly benefits patients with ARDS^60^. Because human alveolus chips can be used to study the effects of applying strain deformations at different amplitudes and frequencies, they could potentially be used in the future to probe the relationship between these parameters and lung innate immunity, and thereby help to provide design criteria to optimize therapeutic strategies to mitigate ventilator-associated lung injury.

This work led to the discovery that production of S100A7 protein and its binding to RAGE mediate the mechanical induction of innate immunity we observed in the alveolus chips. S100A7 also has been shown to exhibit antibacterial and antifungal activities^61,62^. A role for S100A7 in antiviral host responses has not been reported previously; however, it attracted our interest because it has been shown to exert immunomodulatory effects by binding to the inflammation associated RAGE^63,64^. Importantly, RAGE deficiency increases sensitivity to viral infection in animal models^65^, and the closely related S100A8 and S100A9 proteins have been shown to induce innate immune programming and protect newborn infants from sepsis^66^. However, while these S100 proteins help to resolve infections, they can cause severe inflammation and tissue damage when they are aberrantly expressed^67^. For example, S100A8, S100A9, and S100A12 levels in blood have recently been shown to correlate with disease severity in COVID-19 patients infected with SARS-CoV-2^50,51^. S100A7 has been previously shown to exerts its immunomodulatory functions via RAGE-dependent activation of MAPK and NFκB signaling pathways^64^. Interestingly, RAGE is more highly expressed in lung ATI cells at baseline than any other cell type, many of which show little to no RAGE expression^68^, and it appears to play a major role in pulmonary inflammation in various diseases, including COVID-19^68,69^. Our finding that S100A7 expression is controlled mechanically in lung alveoli is also consistent with the observation that it is also induced by mechanical stress in human dental pulp cells^70^. Activation of the S100A7-RAGE pathway may be essential for development of host innate immunity in newborns as well since high amounts of the perinatal alarmins S100A8 and S100A9 have been reported to induce innate immune programming and protect newborn infants from sepsis^66^. While our results demonstrate that S100A7 is sufficient to induce interferon-mediated innate immunity, it is likely not sufficient to explain other effects of mechanical deformation, since both innate immunity and the RAGE pathway may be activated in other ways (e.g., there are many types of stimulatory and inhibitory RAGE ligands). These mechanisms warrant further investigation.

We also found that inhibition of TRPV4 suppresses the induction of S100A7 and downstream innate immune response pathways driven by mechanical strain in lung alveolar and endothelial cells. This is intriguing because TRPV4 also appears to play a similar role in regulate innate responses within immune cells in the lung^7,55,56^. In contrast to inhibition of RAGE, pretreatment of the alveolus chips with a TRPV4 inhibitor decreased viral load despite decreased cytokines as predicted, which is likely due to a separate and more direct effect of TRPV4 signaling on the DDX3X protein, which directly influences virus import into the nucleus and subsequent translation^44^. Therefore, the ultimate effect of TRPV4 inhibition on viral load will depend on relative balance between these opposing activities; however, DDX3X signaling appears to dominate under our experimental conditions. Future research using iPS cell-derived alveolar epithelial cells that are deficient in these genes may offer a better solution to tease out the mechanistic details. Nevertheless, our data suggest that TRPV4 inhibitors may represent an attractive therapy for viral pneumonia as they may both suppress viral load via DDX3X signaling and dampen the associated inflammatory response via the S100A7/RAGE pathway.

Finally, we demonstrated that influenza virus infection also induces the expression of S100A7, S100A8, S100A9, and S100A12 in the alveolus chip, a finding that is consistent with recent clinical observations from COVID-19 patients that exhibit late stage lung infections with SARS-CoV-2 virus^29,50,51,71–73^. While the innate immune response mediated by the S100 alarmins may be beneficial in combating against initial infection, sustained expression and dysregulation of alarmins can result in hyperinflammatory responses that cause irreversible tissue damage, as encountered by patients with severe diseases that require mechanical ventilation^74^. Importantly, using a clinical-relevant dosing strategy in the alveolus chips (i.e., by perfusing them with drugs at their clinical *C*_max_), we found that administration of RAGE inhibitor drugs, such as azeliragon and FPS-ZM1, inhibit viral-induced secretion of inflammatory cytokines when used alone and these effects are amplified when azeliragon is administered therapeutically (2 hours after infection) in combination with the antiviral drug molnupiravir. As azeliragon was found to be well tolerated in phase III trials, it represents an attractive treatment option for attenuating aberrant host immune responses due to viral infection and/or mechanical ventilation in patients with pneumonia or ARDS. But the timing of dosing should be examined with caution because inhibiting RAGE activity during early stage of lung infections may shut off host anti-viral responses and lead to prolonged viral clearance, as suggested by our prophylactic treatment with high dose azeliragon (Fig. 7c). However, it is important to note that a recent study showed that treatment of mice infected with SARS-CoV-2 with the RAGE inhibitor (FPS-ZM1) that we showed suppresses cytokine production in response to influenza virus infection, significantly increased animal survival and limited disease pathogenesis whether administered before or after viral infection^75^. Thus, RAGE inhibition can be a clinically relevant therapeutic target in viral pneumonia and RAGE inhibitor drugs can be used safely in the context of infection with a respiratory virus. Nevertheless, future research will also need to be carried out to define the optimal therapeutic window for use of RAGE and TRPV4 inhibitors in patients with viral infection.

Our study also has multiple limitations. First, as an in vitro model, the human alveolus chip cannot replace animal infection models to evaluate the impact of new therapies on IAV-induced weight-loss or mortality; however, the cellular and molecular features underlying these global manifestations are easily probed using our model, such as barrier loss, cell apoptosis, and activation of innate and adaptive immune responses. Second, the current model does not include alveolar macrophages, which also can contribute significantly to antiviral immune responses. But due to the modular feature of the alveolus chip, primary alveolar or monocyte-derived macrophages can be integrated into the epithelial channel of the chip, as recently demonstrated in a murine lung-on-chip model^76^. This approach could be used, for example, to determine how cyclic mechanical deformations affect macrophage activation and other inflammatory activities in the future. Additionally, because the air channel is accessible to the incubator with a 20% oxygen concentration and PDMS is permeable to air, the oxygen concentration remains constant under all experimental settings. As a result, our current model precludes exploration of the effect of oxygen concentration on innate immunity and viral infection. Also, as we only model transverse deformations of the cells and tissues in parallel to the alveolar-capillary interface in our chip, and the PDMS membrane is significantly stiffer than the tissues, we cannot easily study effects of surfactant-related changes in surface tension or ventilation-related transepithelial pressure changes and anisotropic stains in the current model^77^. It also should be noted that in our studies using alveolus chips lined by epithelial cells from different human donors, we found that azeliragon did not block all cytokines to the same level in every donor (e.g., RANTES effects in Fig. 8f versus 8c); however, the overall trend of inducing suppression of inflammatory cytokines was consistent across multiple donors.

As demonstrated by the current COVID-19 pandemic, potential pandemic viruses, including influenza A viruses, pose a major threat to public health. The lack of experimental models of the distal lung has greatly handicapped our efforts to repurpose existing drugs, understand disease pathology, and develop new therapies. Using influenza A virus as an example, we extend past work focused on viral infection of large airway^27^ by demonstrating that the alveolus chip recapitulates viral receptor expression, strain-dependent infectivity, and system-level host responses in the alveolar epithelial, endothelial, and immune cells during infection by viruses that target alveoli. These features have been overlooked in previous influenza research using cell lines or animal models, but are indispensable for better understanding of disease pathogenesis and identification of more predictive biomarkers and effective therapeutic targets. Thus, in addition to serving as a model system for gaining greater insight into mechanochemical mechanisms that underlie this form of innate immunity, it might serve as a useful preclinical model for identification and optimization of new and more effective antiviral therapeutics for diseases of the distal airway that lead to alveolitis and ARDS. In this context, it is interesting to note that data from this manuscript were recently included in an Investigation New Drug (IND) application to the FDA requesting entry of azeliragon into human clinical trials for treatment of viral pneumonia in hospitalized COVID-19 patients.

## Methods

### Alveolus chip culture

Microfluidic two-channel Organ Chip devices and automated ZOE instruments used to culture them were purchased from Emulate Inc (Boston, MA, USA). After chip activation using ER1/ER2 reagents following manufacturer’s instruction, both channels were seeded with 200 µg/ml Collagen IV (5022-5MG, Advanced Biomatrix) and 15 µg/ml of laminin (L4544-100 UL, Sigma) at 37 °C overnight. The next day (day 1), primary human lung microvascular endothelial cells (Lonza, CC-2527; obtained at P3 and expanded according to manufacturer’s instruction to P5 before use) and primary human lung alveolar epithelial cells (Cell Biologics, P1, H-6053, Lot # F101517Y72; used after thawing without further expansion) were sequentially seeded in the bottom and top channels of the chip at a density of 8 × 10^6^ and 1.6 × 10^6^ cells/ml, respectively, under static conditions. On day 2, the chips were inserted into Pods (Emulate Inc.), placed within the ZOE instrument, and the apical and basal channels were respectively perfused (60 µL/h) with epithelial growth medium (Cell Biologics, H6621) and endothelial growth medium (Lonza, CC-3202). On day 5, 1 µM dexamethasone was added to the apical medium to enhance barrier function. On day 7, an air-liquid interface (ALI) was established in the epithelial channel by flowing at 1000 μL/h for 5 min until all medium from this channel was emptied. Chips were fed through the lower vascular channel, and this medium was changed to EGM-2MV with 0.5% FBS on day 9. Two days later, the ZOE instrument was used to apply cyclic (0.25 Hz) 5% mechanical strain to the engineered alveolar-capillary interface to mimic lung breathing on-chip. Chips were used for experiments on Day 15. In experiments included in Figs. 2c–g, 3h, and 8d, e, the alveolar epithelial cells were isolated from an alternative donor source as previously described^78^. The donor tissue was obtained from the International Institute for the Advancement of Medicine (IIAM) and used in accordance with the regulations by IIAM. All uses of human material have been approved by the Harvard Longwood Campus Institutional Review Board.

### Viral stocks

Influenza virus strains used in this study include A/WSN/33 (H1N1), A/Hong Kong/8/68/ (H3N2), and A/Hong Kong/156/1997 (H5N1). All viruses were obtained from the Centers for Disease Control and Prevention (CDC) or kindly shared by Drs. P. Palese, R.A.M. Fouchier, and A. Carcia-Sastre. Influenza virus strains were expanded in MDCK.2 cell^79^. OC43 coronavirus (VR-1558) was obtained from the ATCC and expanded in HCT-8 cells (ATCC, #HRT-18) as previously described^80^.

### Viral infection on chip

Chips and pods were removed from ZOE and put in biosafety cabinet (BSC). Chips were disconnected from pods and 40 µl viral inoculate was added to the top channel inlet to infect alveolar epithelial cells. Then chips were put back to pods and kept in static condition at 37 °C. Two hours later, chips were removed from pods and top channels were washed with 100 µl DPBS (−/−). Chips were reprimed, connected to pods and continued for flow at 60 µL/h and 5% mechanical strain.

### RNA extraction from Chip for RNA-seq

For RNA extraction, chips were removed from pods. An empty 200 µL filtered tip was inserted to top channel outlet and washed with 100 µL DPBS (+/+) into the top channel inlet to wash the apical channel. After collecting top channel washes, a new empty 200 µL filtered tip was inserted to top channel outlet and 100 µl RNase easy lysis buffer (Qiagen, #74034) was used to lyse epithelial cells by quickly pressing and releasing the micropipette plunger three times. Lysates were collected in a clean labeled 1.5 ml tube and stored at −80 °C for RNA-seq analysis. Endothelial cell lysates were subsequently collected in a similar manner.

### RNA-seq and bioinformatic analysis

RNA-seq was performed by Genewiz using a standard RNA-seq package that includes polyA selection and sequencing on an Illumina HiSeq with 150-bp pair-ended reads. Sequence reads were trimmed to remove possible adapter sequences and nucleotides with poor quality using Trimmomatic v.0.36. The trimmed reads were mapped to the Homo sapiens GRCh38 reference genome using the STAR aligner v.2.5.2b. Unique gene hit counts were calculated by using feature Counts from the Subread package v.1.5.2 followed by differential expression analysis using DESeq2. Gene Ontology analysis was performed using Database for Annotation, Visualization and Integrated Discovery (DAVID, version 6.8)^81^ and the Enrichplot R package (version 1.14.2). Volcano plots were generated using the EnhancedVolcano R package (version 1.6.0). Heatmaps and scatter plots were generated using the ggplot2 R package (version 3.3.2). Short time-series expression miner (STEM, version 1.3.13)^17^ was applied to identify significant temporal patterns using the expression profiles of differentially expressed genes. Five significant patterns were identified with *p*-value < 0.01 and cluster size >300 genes.

### RT-qPCR

Total RNA was isolated using the RNeasy Mini plus Kit or the RNeasy Micro plus Kit (Qiagen). After determining RNA concentrations by spectrophotometry, 50–500 ng of total RNA was used for cDNA synthesis. Reverse transcription was conducted using the Omniscript RT Kit (Qiagen) or the Sensiscript RT kit (Qiagen). Quantitative real-time PCR was performed using the SsoAdvanced- Universal SYBR Green Supermix (Biorad). The specificity of primers was confirmed by melting curve analysis and gel electrophoresis. qPCR was performed on a CFX Connect Real Time PCR Detection System (Biorad). Relative RNA level was quantified using the ΔΔCt method^82^ and normalized to the endogenous control GAPDH unless specified otherwise. All primers were purchased from IDT (Supplementary Table 1).

### PBMC study on chip

At 23 h after infection, PBMC was prepared for the adhesion study. PBMC (StemCell, #70025.1) was thawed and resuspend in 10 ml DMEM with 10% FBS and 1% Penicillin Streptomycin. 10 µl cell tracker green at a final concentration of 10 µM (Invitrogen, #C7025) was used to label cells for 20 min at 37 °C. Then cells were centrifuged and resuspend to 5  × 10^7^/ml. At 24 h after infection, chips were detached from pods and 25 µl labeled PBMC were added to the bottom channel. Chips were flipped and placed on chip cradles. PBMC were allowed to adhere at 37 °C for 2 h before flipped back and washed with 100 µl flow media to wash out unattached cells. Chips were immediately imaged under a fluorescent microscope.

For experiments to determine the relative percentages of different types of immune cells recruited on the chip, apheresis collars (a by-product of platelet isolation) were obtained from Brigham and Women’s Hospital under the approval obtained from the Institution Review Board at Harvard University. PBMCs were isolated by density centrifugation using Lymphoprep (StemCell Technologies, #07801), and magnetic beads were used for negative selection of bulk B cells (StemCell Technologies, #19054), T cells (#17951) and monocytes (#19058). Then B cells were stained with CellTracker Deep Red (C34565), T cells with CellTracker Green CMFDA (C7025), monocytes with CellTracker Violet BMQC (C10094), which were subsequently mixed by the ratio 1:3:6 (B Cells: T cells: monocytes). The mixture was then added to the basal channel as the bulk PBMC experiment described. Cells were counted using the Cell Counter plugin in Image J.

### Flow cytometry

Mixed immune cells were analyzed using an CytoFlex LX (Beckman coulter), and data were analyzed using FlowJo V10 software (Flowjo, LLC). Cell viability was evaluated using ViaKrome 808 Fixable Viability Dye (1:100 dilution; beckman coulter, C36628). The list of antibodies used for flow cytometry can be found in the antibody list (Supplementary Table 2).

### Immunostaining and confocal microscopy

Cells were rinsed with PBS(−/−), fixed with 4% paraformaldehyde (Alfa Aesar) for 20 min at room temperature, permeabilized with 0.1% Triton X-100 (Sigma-Aldrich) in PBS (PBSX) for 10 min, blocked with 5% goat serum (Life Technologies, #50062Z) in PBSX for 1 h at room temperature, and incubated with antibody diluted in blocking buffer (5% goat serum in PBSX) overnight at 4 °C, followed by incubation with fluorescent-conjugated secondary antibody for 1 h at room temperature; nuclei were stained with DAPI (Invitrogen) after secondary antibody staining. Cell apoptosis/ necrosis assay kit (Abcam, #ab176749) was used to quantification cell death according to manufacturer’s instructions. Immunostaining of Ki67 (Abcam, # ab15580) was used to measure cell proliferation. Fluorescence imaging was conducted using a confocal laser-scanning microscope (SP5 X MP DMI-6000, Germany) and image processing was done using the ImageJ software (version 2.1.0). DAPI (4′,6-Diamidino-2-Phenylindole, Dihydrochloride) was purchased from Invitrogen (#D1306). The list of antibodies used for immunostaining can be found in the antibody list (Supplementary Table 2).

### Permeability assay

Prior to the assay, both the inlet and outlet reservoirs were emptied. Cascade blue (Life Technologies, # C687) and 3kD Texas red-dextran (Life Technologies, # D3329) were diluted in the flow medium to final concentrations of 100 µg/ml for both tracers, and 500 µl of the mixture was added to the bottom channel inlet reservoir, while 500 µl of the flow medium without the tracers was added to the top channel inlet reservoir. The chips were then flushed for 5 min at a rate of 600 µL/h to allow the dosing solution to fill the Pod and prime the channels. The chips were then flowed at 120 µl/h for both channels for 2 h before collecting medium from the outlet reservoirs and measuring the fluorescence absorbance. The apparent permeability was calculated as previously described^36^.

### Cytokine analysis

Vascular effluents from alveolus chips were collected and analyzed for a panel of cytokines and chemokines, including IL-6, IL-8, IP-10, TNF, RANTES, S100A8/A9, and GM-CSF using custom ProcartaPlex assay kits (Invitrogen). Analyte concentrations were determined using a Luminex100/200 Flexmap3D instrument coupled with Luminex XPONENT software (Luminex, USA, version 4.2). S100A7 was measured using an ELISA kit (Aviva Systems Biology) according to manufacturer’s instruction.

### Cytotoxicity assay

Vascular outflows from the alveolus chip were collected 48 h after pretreatment with TRPV4 or RAGE inhibitor. The amount of lactate dehydrogenase (LDH) was measured as a readout for cytotoxicity using the LDH-Glo Cytotoxicity Assay (Promega, # J2380) following manufacturer’s instruction.

### Drug treatment on-chip

Azeliragon (TTP-488) and FPS-ZM1 were purchased from MedChemExpress (#HY-50682) and Sigma (#553030), respectively. Molnupiravir (EIDD-1931) was purchased from Cayman Chemical (#9002958). TRPV4 inhibitor GSK2193874 was purchased from Tocris Bioscience (#No. 5106). Drugs were diluted using alveolus chip flow media with a final DMSO concentration of 0.5% and perfused at 30 µl/h through the vascular channel prophylactically or therapeutically at the time points and doses indicated in each figure.

### Culture and transfection of A549 cells

A549 cells (ATCC CCL-185) were cultured in Dulbecco’s modified Eagle’s medium (DMEM) (Life Technologies) supplemented with 10% fetal bovine serum (FBS) (Life Technologies) and penicillin-streptomycin (Life Technologies). All cells were maintained at 37 °C and 5% CO_2_ in a humidified incubator. Transfection was performed using TransIT-X2 Dynamic Delivery System (Mirus) according to the manufacturer’s instructions. pCMV6-XL5 empty plasmid (PCMV6XL5) and S100A7 plasmid were purchased from Origene (#SC122639). RAGE plasmid was purchased from Sino Biological (HG11629-ACG). Endotoxin-free plasmid purification was performed by Genewiz.

### Culture and transfection of human primary alveolar epithelial cells

Primary human lung alveolar epithelial cells (Cell Biologics, H-6053) were seeded on 0.1% gelatin-coated 12-well plate according to manufacturer’s instruction. When reaching 70% confluent, cells were transfected with pCMV6-XL5 empty plasmid or S100A7 plasmid using TransIT-X2 Dynamic Delivery System (Mirus).

### Transfection in human alveolus chip

Human alveolus chips were transfected with plasmid DNA using the TransIT-X2 reagent (Mirus Bio). In all, 3 μg of plasmid DNA and 6 μl TransIT-X2 reagent were constituted in 300 μl OPT-MEM before added to 3 ml chip flow medium. Then 1.5 ml of the solution was added to apical and basal inlet reservoirs and flew at 60 μl/h for 24 h. Then apical channel was emptied to reestablish ALI; basal channel was changed to normal flow medium for additional 24 h before qPCR analysis.

### Plaque assay

Virus titers were determined by plaque assay. Confluent MDCK cell monolayers in 12-well plate were washed with PBS, inoculated with 1 mL of 10-fold serial dilutions of influenza virus samples for 1 h at 37 °C, and then overlaid with 1 mL of DMEM (Gibco) supplemented with 1.5% low melting point agarose (Sigma-Aldrich) and 2 µg/mL TPCK-treated trypsin (Sigma-Aldrich). 2–4 days after incubation at 37 °C under 5% CO_2_, the cells were fixed with 4% paraformaldehyde (Alfa Aesar) and stained with crystal violet (Sigma-Aldrich) to visualize the plaques; virus titers were determined as plaque-forming units per milliliter (PFU/mL).

### Statistics

Each experiment was repeated at least three times, with at least three biological replicates for each data point. Data are displayed as mean values ± standard deviation (SD) unless otherwise noted in the figure legends. Graphing and statistical comparison of the data were performed using Prism 9.2.0 (GraphPad Software) and Excel v16.53 (Microsoft). Unless otherwise noted, two-group comparisons were assessed using the two-tailed Student’s t test; comparison of three or more groups were analyzed by one-way ANOVA with Bonferroni multiple comparisons for pairwise comparisons and Dunnett’s test for comparisons involving a single control group. Images represent at least two independent experiments for immunofluorescence images shown in Figs. 1b, d, h and 2a, b; supplementary figs. 1b and 6e. P values < 0.05 were considered to be statistically significant; exact *p* values are labeled in each figure.

### Reporting summary

Further information on research design is available in the Nature Research Reporting Summary linked to this article.

## Supplementary information

Supplementary Information

Description for Additional Supplementary Files

Supplementary Data 1

Supplementary Data 2

Supplementary Data 3

Supplementary Data 4

Reporting Summary

## Data Availability

RNA-sequencing data have been deposited in the Gene Expression Omnibus (GEO) database under the accession code GSE177862, GSE178266, and GSE178382. Flow cytometry data has been deposited in the Flow Repository with the accession ID FR-FCM-Z52C. Additional data are presented in the Supplementary Materials. There are no restrictions on data availability in the current work. Source data are provided with this paper.

## Source data

Source Data

## Code availability

The code for bulk RNA-sequencing data analysis was based on the official tutorials of the packages listed in Methods. No other custom code was used for the creation of this manuscript.
