# Supplementary material for: Mechanical control of innate immune responses against viral infection revealed in a human lung alveolus chip
Source: Nat Commun. 2022 Apr 8;13:1928. doi: 10.1038/s41467-022-29562-4 (PMC8993817; doi:10.1038/s41467-022-29562-4)
Supplement: Supplementary file 1 — Supplementary Information [file 41467_2022_29562_MOESM1_ESM.pdf]

SUPPLEMENTARY INFORMATION

Mechanical control of innate immune responses against viral infection revealed in a human Lung Alveolus Chip

Haiqing Bai[1], …, and Donald E. Ingber[1-3]

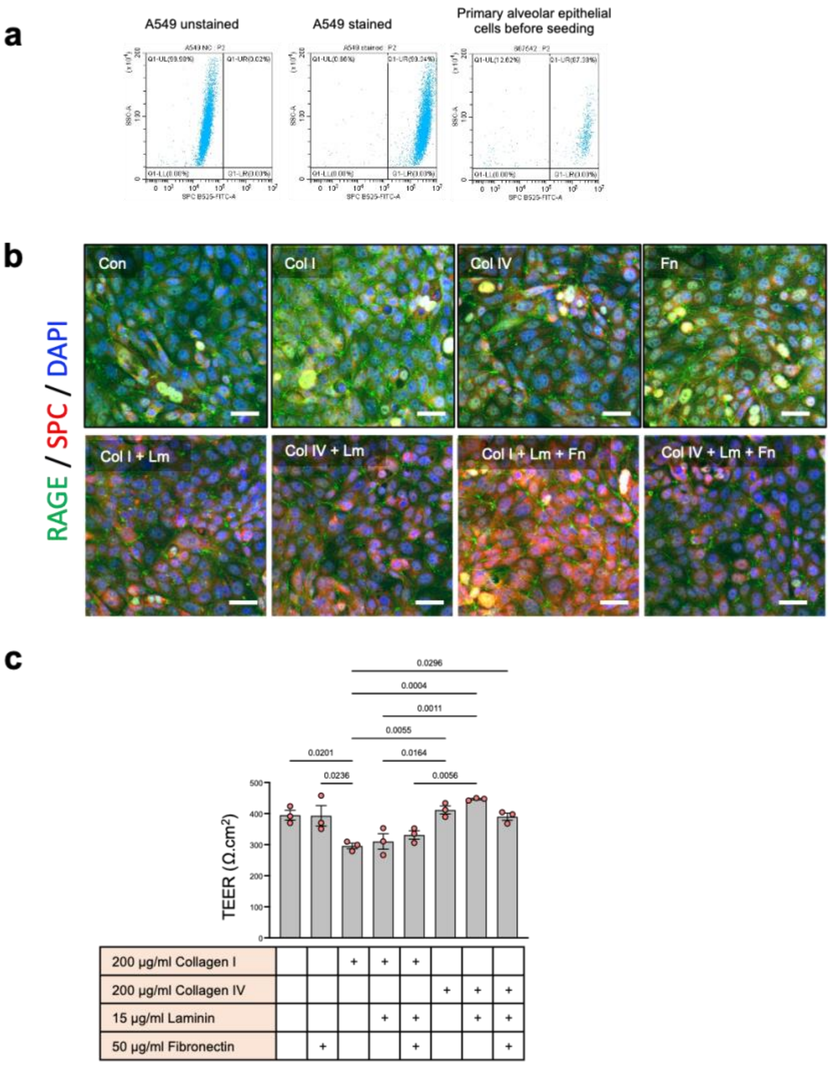

Supplementary Figure 1. Protocol optimization for culture of human Alveolus Chip. (**a**) Flow cytometry showing 87% cells seeded onto chip are positive stained with type II marker SPC. A549 was used as a positive control for staining. (**b**) Immunofluorescence micrographs showing the expression levels and distribution of ATI cell marker RAGE and ATII cell marker SPC type II in the epithelium of Alveolus Chips coated with different ECM components. Con, without ECM; Col I, Collagen I; Col IV, Collagen IV; Fn, Fibronectin; Lm, Laminin. Scale bar, 50 µm. (**c**) The effects of different ECM coatings on barrier function as measured by transepithelial electrical resistance (TEER). Data are shown as mean ± SD; n = 3 biological replicates; unpaired two-tailed t-test (compared with no coating control). Source data are provided as a Source Data file.

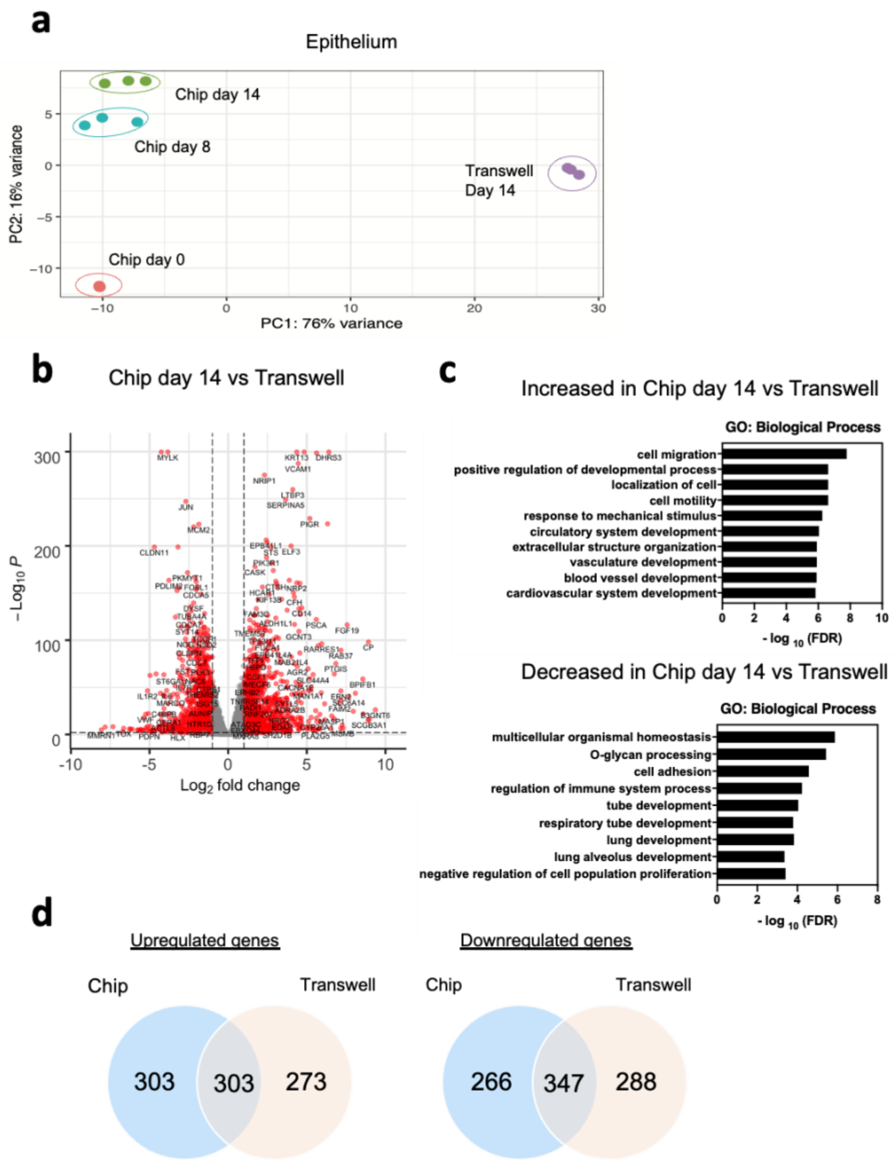

Supplementary Figure 2. Characterization of Alveolus Chips and comparison with Transwell culture. (**a**) PCA plot of RNA-seq datasets from the Alveolus Chip or the Transwell. (**b**) Volcano plot of DEGs comparing epithelial cells from Alveolus Chips at day 14 of culture with Transwell culture. DEGs ($P_{adj} < 0.01$) with a fold change >2 (or <−2) are indicated in red. The names of top DEGs are shown. P values were adjusted using Bonferroni correction for multiple comparisons. (**c**) Gene ontology analysis showing enriched biological processes from (**b**). (**d**) Venn diagram to show the numbers of common and unique gene regulation in epithelial cells of Alveolus Chip and the Transwell culture.

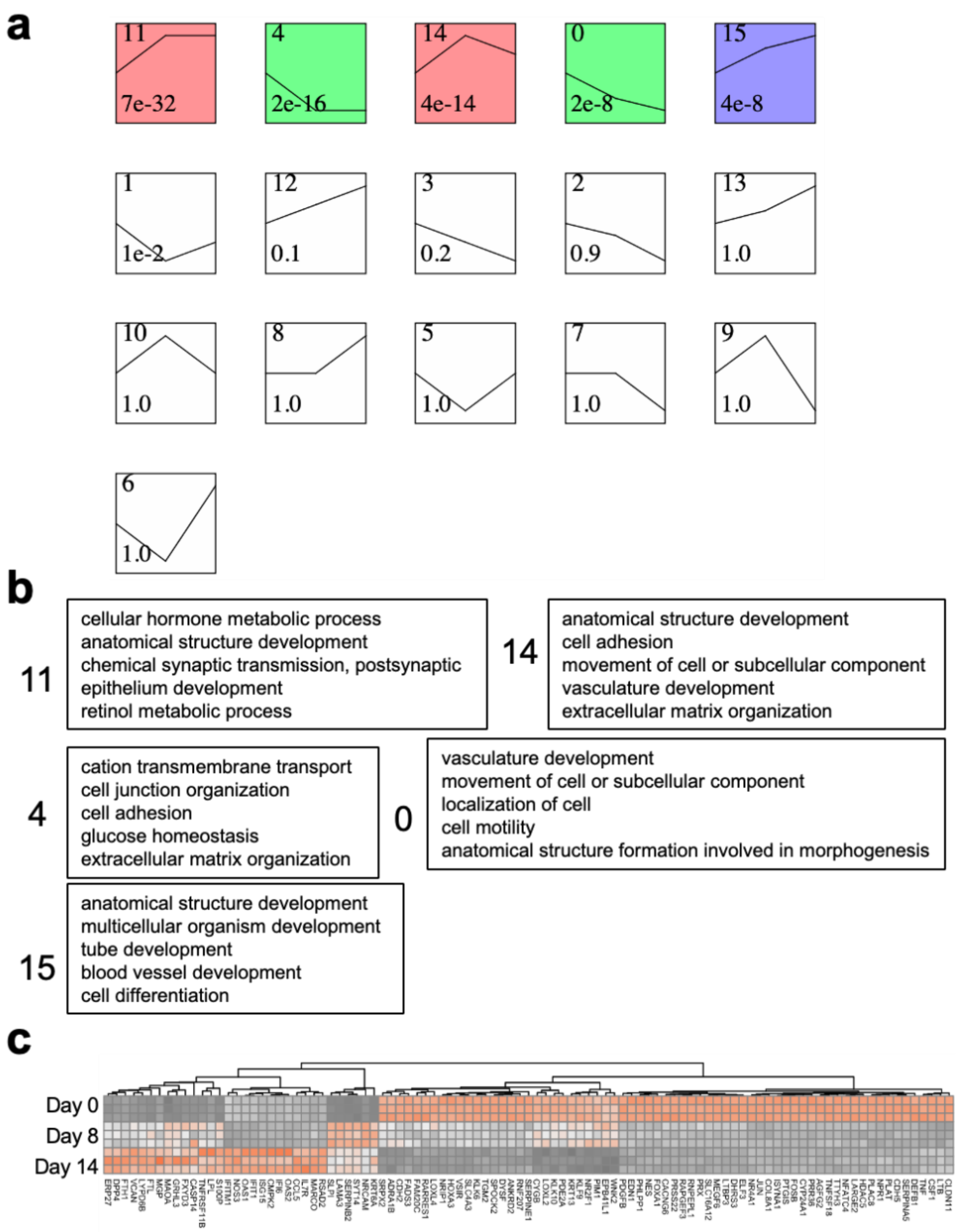

Supplementary Figure 3. Temporal gene expression patterns in epithelial cells of Alveolus Chip. (**a**) All gene expression patterns identified using short time-series expression miner (STEM). Significant patterns were colored and p value was shown inside the box. (**b**) Functional annotations enriched by the temporal gene expression patterns that are significant in (**a**). (**c**) Heat map showing the top differentially expressed genes in epithelial cells on-chip at day 0, 8 and 18 of culture.

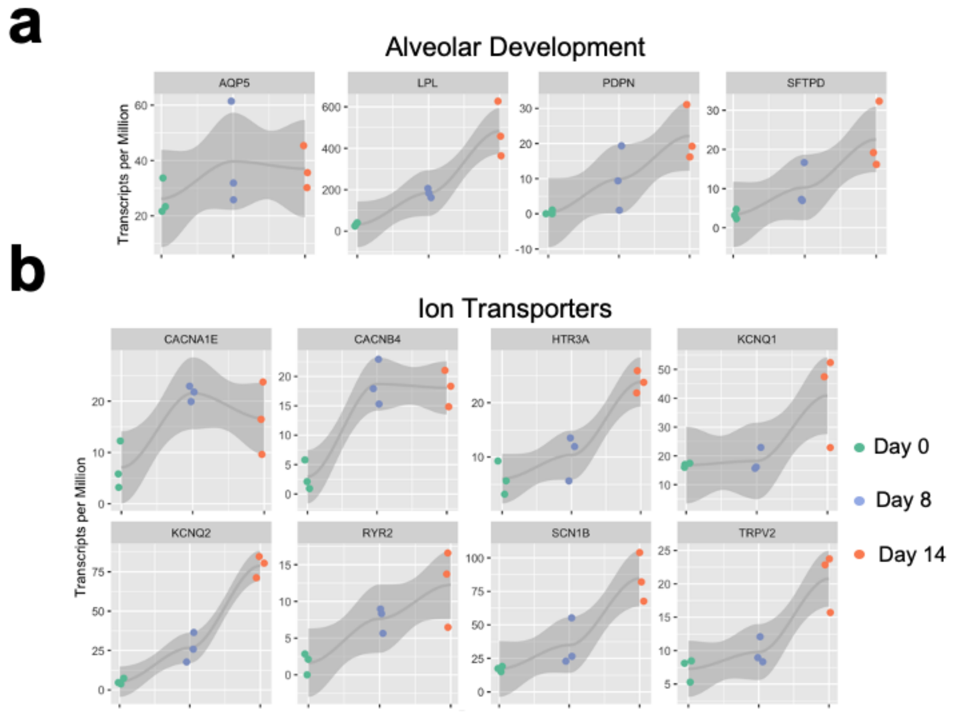

Supplementary Figure 4. Time course of expression of selected genes that are involved in (**a**) alveolar development and (**b**) ion-transport in the epithelial cells in the Alveolus Chip. Grey zone indicates the 95% confidence interval for predications from a linear model.

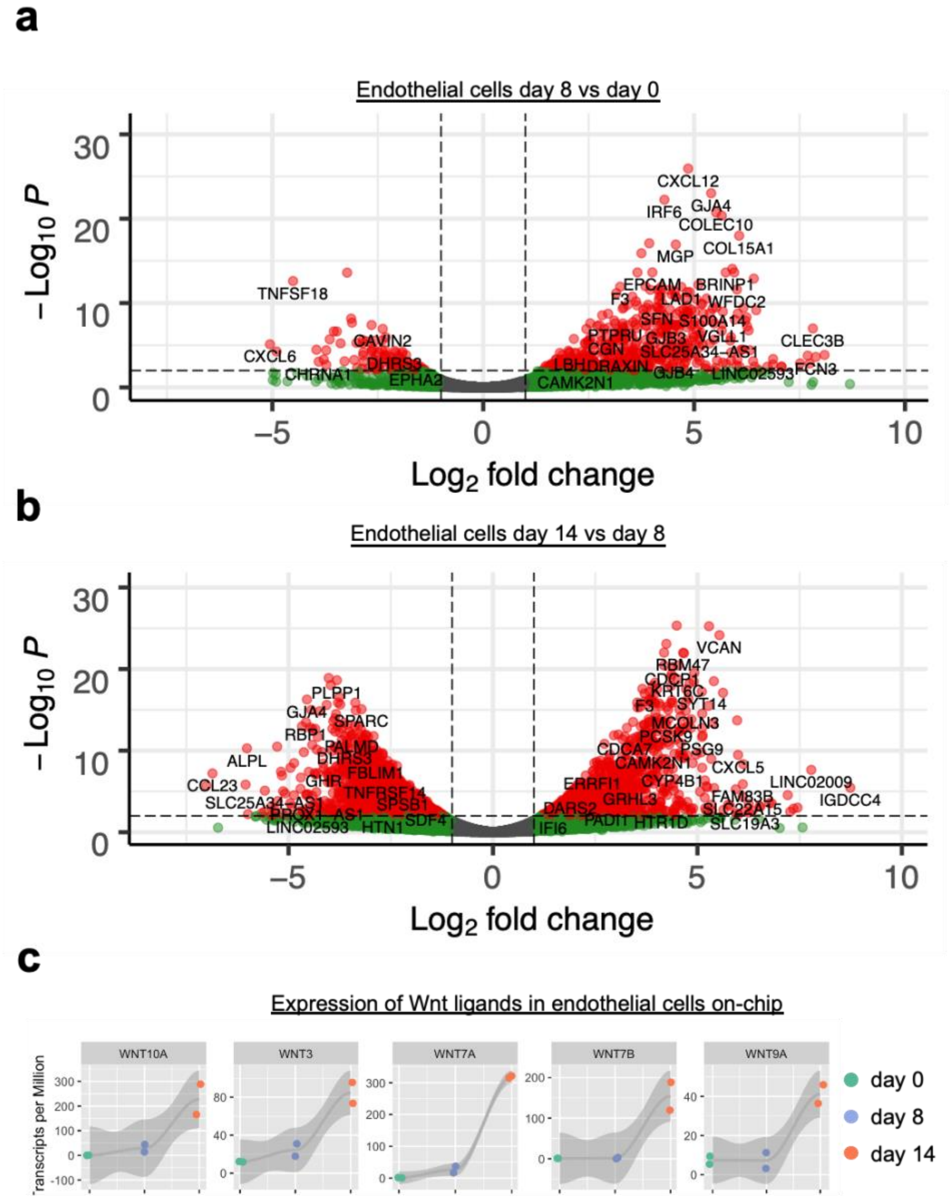

Supplementary Figure 5. Transcriptomic changes in endothelial cells of Alveolus Chip. (a) Volcano plot of DEGs showing the transcriptomic changes in endothelial cells of Alveolus Chips at day 8 vs day 0 of culture. (**b**) Volcano plot of DEGs showing the transcriptomic changes in endothelial cells of Alveolus Chips at day 14 vs day 8 of culture. P values were adjusted using Bonferroni correction for multiple comparisons for **a** and **b**. (**c**) Increased expression of several Wnt ligands in endothelial cells on-chip during Alveolus Chip maturation. Grey zone indicates the 95% confidence interval for predications from a linear model.

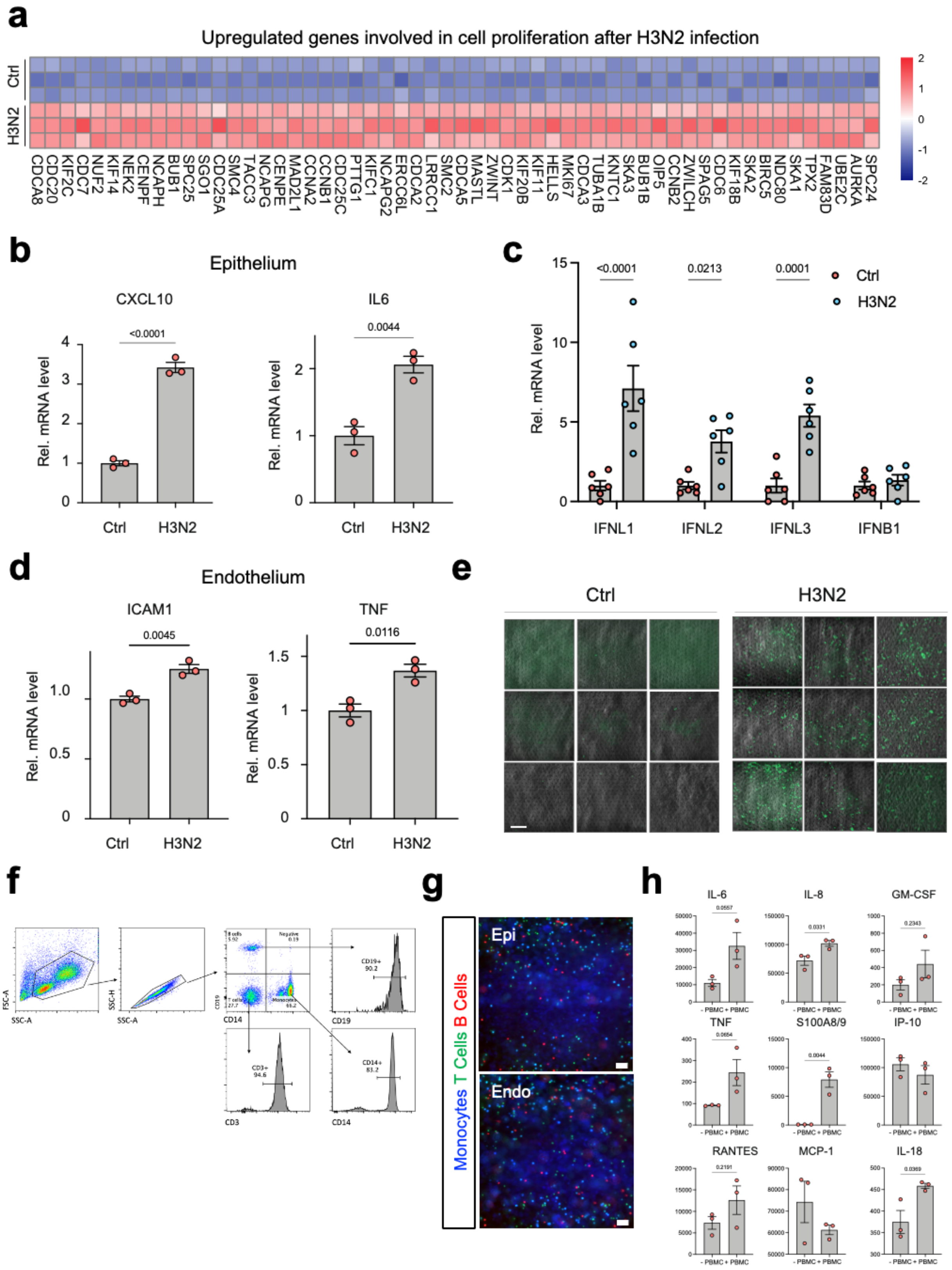

a
Upregulated genes involved in cell proliferation after H3N2 infection
Ctrl
H3N2
CDCA8 CDC20 KIF2C CDC7 NUF2 KIF14 NEK2 CENPF NCAPH BUB1 SPC25 SGO1 CDC25A SMC4 TACC3 NCAPG CENPE MAD2L1 CCNA2 CCNB1 CDC25C PTTG1 KIFC1 NCAPG2 ERCC6L CDCA2 LRRCC1 SMC2 CDCA5 MASTL ZWINT CDK1 KIF20B KIF11 HELLS MKI67 CDCA3 TUBA1B KNTC1 SKA3 BUB1B OIP5 CCNB2 ZWILCH SPAG5 CDC6 KIF18B SKA2 BIRC5 NDC80 SKA1 TPX2 FAM83D UBE2C AURKA SPC24
b
Epithelium
CXCL10
IL6
Rel. mRNA level
<0.0001
0.0044
Ctrl
H3N2
c
<0.0001
0.0213
0.0001
Ctrl
H3N2
Rel. mRNA level
IFNL1
IFNL2
IFNL3
IFNB1
d
Endothelium
ICAM1
TNF
0.0045
0.0116
Rel. mRNA level
Ctrl
H3N2
e
Ctrl
H3N2
f
SSC-A
FSC-A
SSC-H
CD14
CD19
CD3
g
Epi
Endo
Monocytes T Cells B Cells
h
IL-6
IL-8
GM-CSF
TNF
S100A8/9
IP-10
RANTES
MCP-1
IL-18
- PBMC + PBMC

Supplementary Figure 6. Host immune responses to influenza infection in Alveolus Chip. (**a**) Heat map showing a list of genes involved in cell proliferation that alter their expression after H3N2 infection on-chip compared with uninfected control (Ctrl). (**b**) Relative mRNA levels of CXCL10 and IL6 in epithelial cells of Alveolus Chips uninfected (Ctrl) and infected with HK/68 (H3N2) (MOI = 1), measured by qPCR at 48 h post-infection. Data are shown as mean ± SD; n = 3 biological replicates; unpaired two-tailed t-test. (**c**) Relative mRNA levels of IFNL1, IFNL2, IFNL3, and IFNB1 in epithelial cells of Alveolus Chips uninfected (Ctrl) and infected with HK/68 (H3N2) (MOI = 1), measured by qPCR at 48 h post-infection. Data are shown as mean ± SD; n = 6 biological replicates; unpaired two-tailed t-test. (**d**) Relative mRNA levels of ICAM1 and TNF in endothelial cells of Alveolus Chips uninfected (Ctrl) and infected with HK/68 (H3N2) (MOI = 1), measured by qPCR at 48 h post-infection. Data are shown as mean ± SD; n = 3 biological replicates; unpaired two-tailed t-test. (**e**) Images showing the adhesion of CellTracker Green-labeled PBMCs to endothelium of Alveolus Chips uninfected (Ctrl) and infected with HK/68 (H3N2). Nine representative images were taken for each condition. Scale bar: 100 µm. (**f**) Flow cytometric analysis of cell purity of magnetically sorted B cells ($CD19^+$), Monocytes ($CD14^+$) and T cells ($CD3^+$) that were individually labeled and perfused through the Alveolus Chip. (**g**) Representative images of B cells, T cells, and monocytes in the epithelial (Epi) or endothelial channel (Endo) of the chip 2 hours after perfusion through the endothelial channel. Scale bar: 50 µm. (**h**) Concentrations of the indicated cytokines measured by Luminex assay in the effluent of the vascular channels of Alveolus Chips infected with HK/68 (H3N2) virus (MOI = 1) in the absence (-) or presence (+) of PBMC at 24 hours post perfusion through the endothelial channel. Data represents mean ± SD; n = 3 biological replicates; unpaired two-tailed t-test. Source data are provided as a Source Data file.

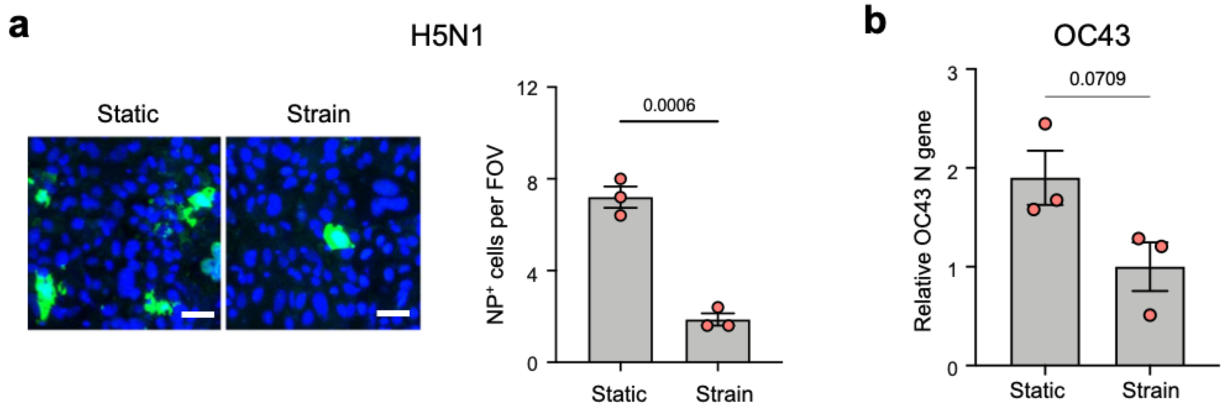

Supplementary Figure 7. Mechanical Strain inhibits infection of the Alveolus Chip by H5N1 influenza virus and OC43 coronavirus. (**a**) Immunofluorescence micrographs (left) showing influenza virus NP (green) in Alveolus Chips that are cultured under static condition (Static) or exposed to physiological cyclic deformations (5% strain, 0.25 Hz) (Strain) for 48 hours and then infected with H5N1 (MOI = 0.001) for another 48 hours (blue, DAPI-stained nuclei; bar, 50 µm). Graph (right) shows the numbers of NP^+ cells per field of view (FOV). Data represent mean ± SD; n = 3 biological replicates; unpaired two-tailed t-test. (**b**) Graph showing relative levels of OC43 N gene measured by qPCR in epithelium within Alveolus Chips that are cultured under static condition (Static) or mechanically stimulated with physiological 5% strain at 0.25 Hz (Strain) for 48 hours and then infected with OC43 (MOI = 5) for another 48 hours. Data represent mean ± SD; n = 3 biological replicates; unpaired two-tailed t-test. Source data are provided as a Source Data file.

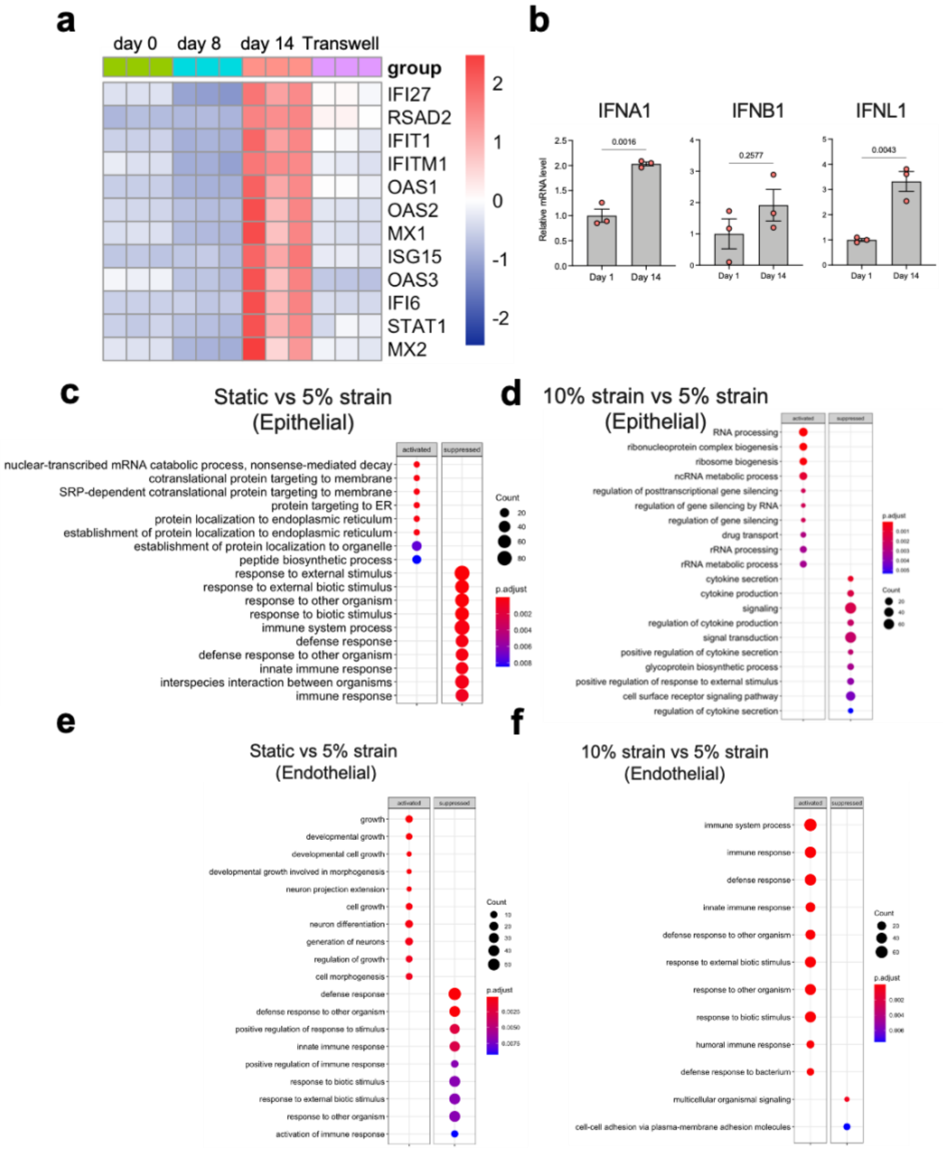

Supplementary Figure 8. Cyclic mechanical strain activates the innate immune responses in Alveolus Chips. (**a**) Heat maps showing expression of interferon stimulated genes (ISGs) in epithelial cells of Alveolus Chips at different time points of culture or in Transwell. (**b**) qPCR analysis showing increased mRNA levels for type I and type III interferons. Data represent mean ± SD; n = 3 biological replicates except for IFNL1 (n = 2); unpaired two-tailed t-test. (**c to f**) Gene ontology (GO) pathway-enrichment analysis of DEGs (**c, d, e, f** corresponds to studies shown in **Fig. 5c, d, f, and g**, respectively. P values were adjusted using Bonferroni correction for multiple comparisons for **c**, **d**, **e**, **f**. Source data are provided as a Source Data file.

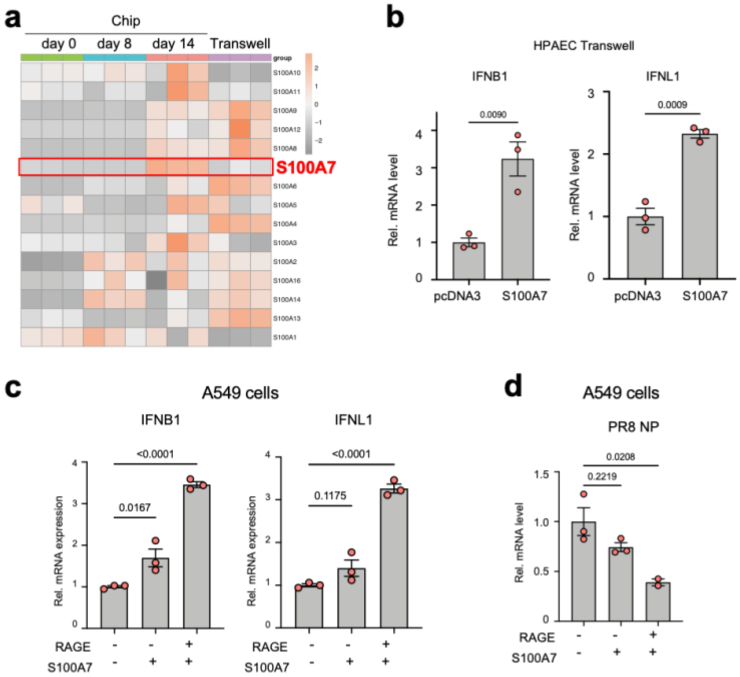

Supplementary Figure 9. S100A7 induces innate immunity in Alveolus Chips. (a) Heatmap showing the specific increase of S100A7 among the S100 family genes during the Alveolus Chip differentiation. (**b**) Graph showing the mRNA levels of IFNβ1 and IFNλ1 in human primary alveolar epithelial cells (HPACE) at 48 hours after transfection with a plasmid expressing S100A7 or the pcDNA3 empty vector control. Data represent mean ± SD.; n = 3 biological replicates; unpaired two-tailed t-test. **(c)** Graph showing the mRNA levels of IFNβ1 and IFNλ1 in A549 cells transfected with a plasmid expressing RAGE for 24 hours and then treated with culture supernatant containing S100A7 for 24 hours. N = 3 biological replicates. Data are shown as mean ± SEM and statistical significance was assessed by one-way ANOVA with Dunnett's multiple comparisons test. (**d**) Graph showing the levels of PR8 influenza virus NP mRNA in A549 cells transfected with a plasmid expressing RAGE for 24 hours, treated with culture supernatant containing S100A7 for 24 hours, and then infected with PR8 (MOI = 0.01) for another 24 hours. N = 2-3 biological replicates. Data are shown as mean ± SEM and statistical significance was assessed by one-way ANOVA with Dunnett's multiple comparisons test. Source data are provided as a Source Data file.

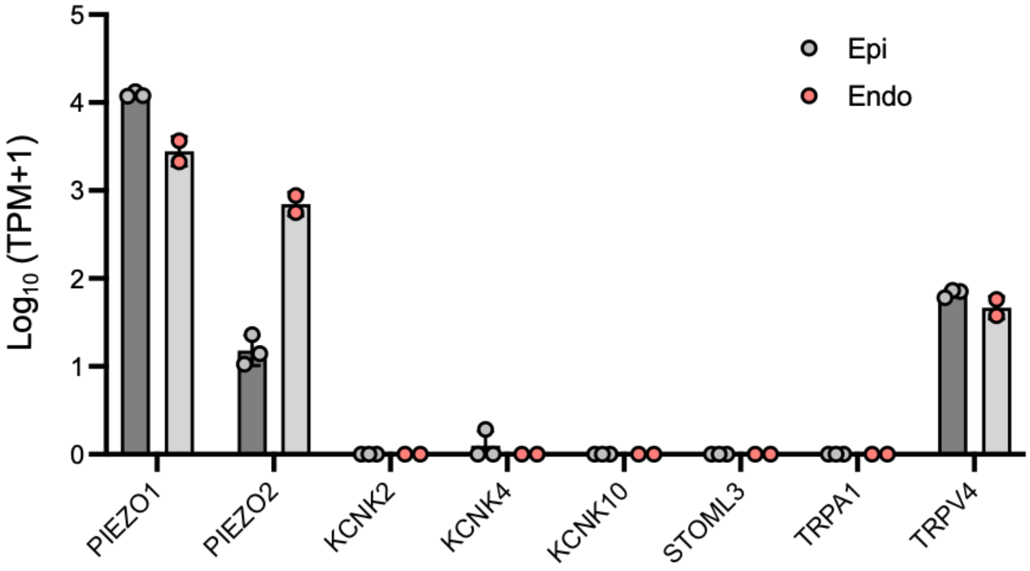

Supplementary Figure 10. Relative abundance of mechanosensitive ion channels expressed in the human Alveolus Chip. Graph shows the expression of these mechanoreceptors in the epithelial and endothelial cells on-chip, as determined from RNA-seq. TPM, transcript per million. Data are shown as mean ± SD and n = 2-3 biological replicates. Source data are provided as a Source Data file.

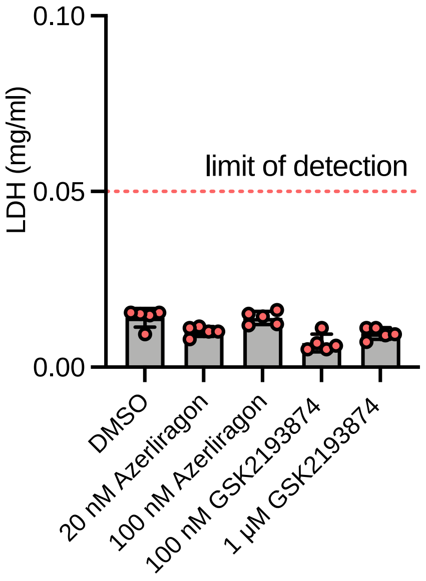

Supplementary Figure 11. Cytotoxicity of TRPV4 inhibitor and RAGE inhibitor on human Alveolus Chip measured using an LDH assay. Data are shown as mean ± SD and n = 5 biological replicates. Source data are provided as a Source Data file.

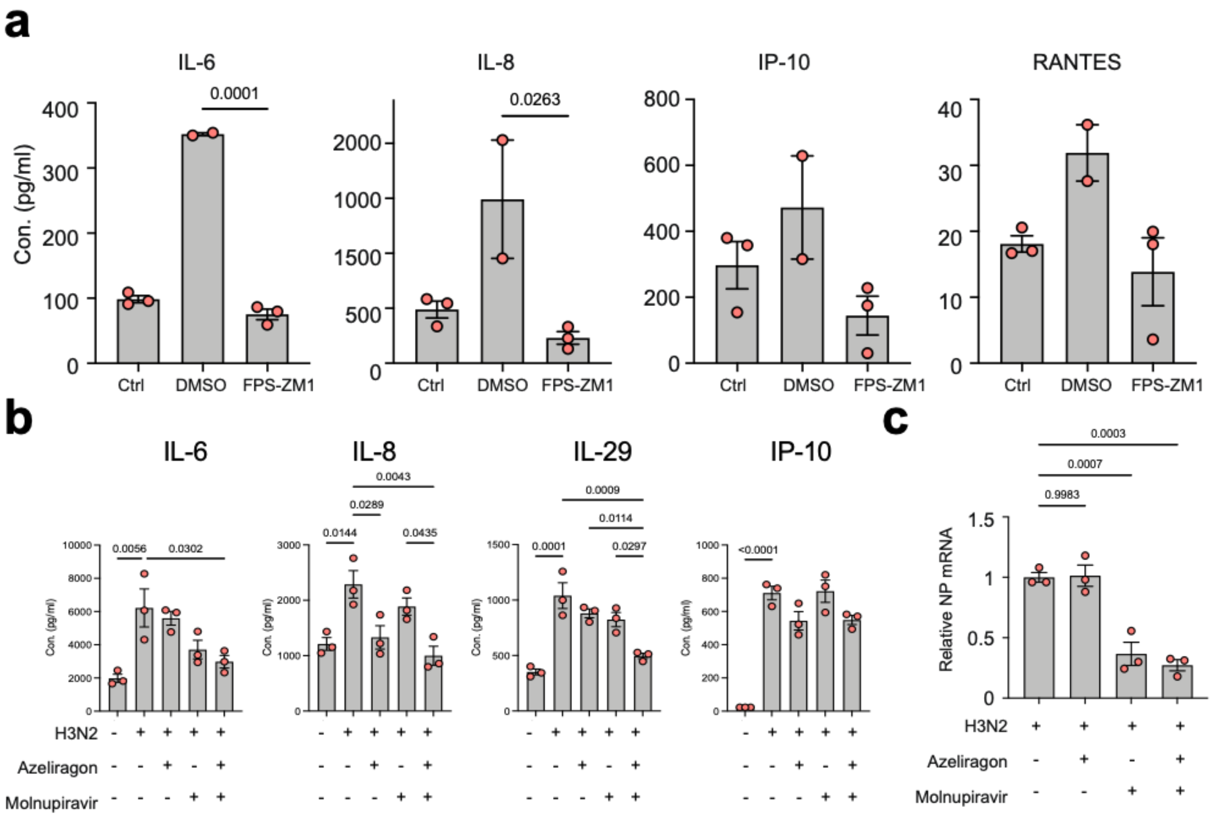

Supplementary Figure 12. RAGE inhibitor suppresses viral host responses (**a**) Graphs showing the levels of cytokines in the vascular effluents of Alveolus Chips that were uninfected (Ctrl) or infected with HK/68 (H3N2) (MOI = 1) in the presence or absence of the RAGE inhibitor FPS-ZM1 (200 nM). Data are shown as mean ± SEM and statistical significance was assessed by one-way ANOVA with Bonferroni multiple comparisons test; n = 3 biological replicates expect for the DMSO group (n=2). (**b**) Graphs showing the levels of cytokines in the vascular effluents of Alveolus Chips that were uninfected (Ctrl) or infected with HK/68 (H3N2) (MOI = 1) in the presence or absence of 100 nM azeliragon, 500 nM molnupiravir or both combined. (**c**) Graph showing mRNA level of H3N2 NP in epithelial cells on-chip when infected with HK/68 (H3N2) (MOI = 1) in the presence or absence of 100 nM azeliragon, 500 nM molnupiravir or both combined. Data are shown as mean ± SEM; n = 3 biological replicates; one-way ANOVA with Bonferroni multiple comparisons test. Source data are provided as a Source Data file.

Supplementary Table 1. Antibody Information

Antigen	Manufacturer	Catalog #	Used in	Dilution
CD3	BD Biosciences	641406	Flow cytometry	1:50
CD14	Biolegend	301806	Flow cytometry	1:50
CD19	Biolegend	302210	Flow cytometry	1:50
proSP-C	Sigma	ab3786	Immunostaining	1:100
Ki67	Abcam	ab15580	Immunostaining	1:200
proSP-C	Sigma	ab3786	Immunostaining	1:200
RAGE	Santa Cruz	sc-365154	Immunostaining	1:200
ZO-1	Invitrogen	MA3-39100-A555	Immunostaining	1:100
VE-cadherin	Invitrogen	14-1449-82	Immunostaining	1:200
MX1	Abcam	ab95926	Immunostaining	1:200
IAV Nuclear protein	Bio X Cell	BE0159-R001MG	Immunostaining	1:200
Mouse IgG-488	Invitrogen	A-11001	Immunostaining	1:1000
Mouse IgG-647	Invitrogen	A-21235	Immunostaining	1:1000
Rabbit IgG-488	Invitrogen	A-11034	Immunostaining	1:1000
Rabbit IgG-647	Invitrogen	A-32733	Immunostaining	1:1000

Supplementary Table 2. qPCR Primer Information

Name	Sequence
GAPDH_F	TGCACCACCAACTGCTTAGC
GAPDH_R	GGCATGGACTGTGGTCATGAG
PDNN_F	CCAGGAACCAGCGAAGACC
PDPN_R	GCGTGGACTGTGCTTTCTGA
SFTPC_F	CACCTGAAACGCCTTCTTATCG
SFTPC_R	TTTCTGGCTCATGTGGAGACC
ICAM1_F	ATGCCCAGACATCTGTGTCC
ICAM1_R	GGGGTCTCTATGCCCAACAA
CXCL10_F	AAATATGGCACACTAGCCCCAC
CXCL10_R	TGGTGCTGAGACTGGAGGTT
CCL2_F	GATCTCAGTGCAGAGGCTCG
CCL2_R	TTTGCTTGTCCAGGTGGTCC
CCL5_F	TCATTGCTACTGCCCTCTGC
CCL5_R	TCGGGTGACAAAGACGACTG
IL6_F	ACTCACCTCTTCAGAACGAATTG
IL6_R	CCATCTTTGGAAGGTTCAGGTTG
TNF_F	CCCATGTTGTAGCAAACCCTC
TNF_R	TATCTCTCAGCTCCACGCCA
SFTPB_F	TGGAGCAAGCATTGCAGTG
SFTPB_R	ACTCTTGGCATAGGTCATCGG
RAGE_F	ACTACCGAGTCCGTGTCTACC
RAGE_R	GGAACACCAGCCGTGAGTT
SFTPD_F	AAGCAGGGGAACATAGGACCT
SFTPD_R	ACACCTCGCTCTCCCTTAGG
S100A7_F	CTCGCCGATGTCTTTGAGAA
S100A7_R	CTTGTGGTAGTCTGTGGCTATG
MX1_F	CGAAACATCTGTGAAAGCAAGC
MX1_R	CAGGCTTTGTGAATTACAGGAC
IFI6_F	GTAGCACAAGAAAAGCGATACC
IFI6_R	CTGCTGTGCCCATCTATCAG
ISG15_F	GCCTTCAGCTCTGACACC
ISG15_R	CGAACTCATCTTTGCCAGTACA
OAS1_F	GATGAGCTTGACATAGATTTGGG
OAS1_R	GGTGGAGTTCGATGTGCTG
IFIT1_F	GCTCCAGACTATCCTTGACCT
IFIT1_R	CCACAAGACAGAATAGCCAGAT
IFNL1_F	GAAGACAGGAGAGCTGCAAC
IFNL1_R	GGTTCAAATCTCTGTCACCACA
