# Supplementary material for: Mechanical control of innate immune responses against viral infection revealed in a human lung alveolus chip
Source: Nat Commun. 2022 Apr 8;13:1928. doi: 10.1038/s41467-022-29562-4 (PMC8993817; doi:10.1038/s41467-022-29562-4)
Supplement: Supplementary file 2 — Description for Additional Supplementary Files [file 41467_2022_29562_MOESM2_ESM.docx]

**Description of Additional Supplementary Files**

File Name: Supplementary Data 1.csv

Description: Differential gene expression analysis for alveolar epithelial cells on Chip day-14 vs day-0.

File Name: Supplementary Data 2.xlsx

Description: Gene Ontology analysis of differentially expressed genes (DEGs) in alveolar epithelial cells on Chip day-14 vs day-0 and on Chip day-14 vs Transwell.

File Name: Supplementary Data 3.xlsx

Description: Comparisons of DEGs on human Alveolus Chip vs on human samples from fetal to birth.

File Name: Supplementary Data 4.xlsx

Description: Calculation of % strain in the human lung under low-tidal volume (6 ml/kg) and high-tidal volume ventilation (12 ml/kg) conditions.
